# Optogenetic regulation of endogenous proteins

**DOI:** 10.1038/s41467-020-14460-4

**Published:** 2020-01-30

**Authors:** Taras A. Redchuk, Maksim M. Karasev, Polina V. Verkhusha, Sara K. Donnelly, Maren Hülsemann, Jori Virtanen, Henna M. Moore, Maria K. Vartiainen, Louis Hodgson, Vladislav V. Verkhusha

**Affiliations:** 1 Medicum, Faculty of Medicine, University of Helsinki, Helsinki, 00290 Finland; 2 Department of Anatomy and Structural Biology, and Gruss-Lipper Biophotonics Center, Albert Einstein College of Medicine, Bronx, NY 10461 USA; 3 Institute of Biotechnology, HiLIFE, University of Helsinki, Helsinki, 00790 Finland; 4 Present Address: Institute of Biotechnology, HiLIFE, University of Helsinki, Helsinki, 00790 Finland

**Keywords:** Optogenetics, Cell signalling, Actin, Synthetic biology

## Abstract

Techniques of protein regulation, such as conditional gene expression, RNA interference, knock-in and knock-out, lack sufficient spatiotemporal accuracy, while optogenetic tools suffer from non-physiological response due to overexpression artifacts. Here we present a near-infrared light-activatable optogenetic system, which combines the specificity and orthogonality of intrabodies with the spatiotemporal precision of optogenetics. We engineer optically-controlled intrabodies to regulate genomically expressed protein targets and validate the possibility to further multiplex protein regulation via dual-wavelength optogenetic control. We apply this system to regulate cytoskeletal and enzymatic functions of two non-tagged endogenous proteins, actin and RAS GTPase, involved in complex functional networks sensitive to perturbations. The optogenetically-enhanced intrabodies allow fast and reversible regulation of both proteins, as well as simultaneous monitoring of RAS signaling with visible-light biosensors, enabling all-optical approach. Growing number of intrabodies should make their incorporation into optogenetic tools the versatile technology to regulate endogenous targets.

## Abstract (web summary)

Optogenetic approaches to control protein-protein interactions usually require overexpression of the target proteins. Here the authors integrate intrabodies into near-infrared- and blue-light activatable optogenetic tools to control endogenous proteins in mammalian cells.

## Introduction

Precise control of protein activity and protein–protein interactions is in high demand. Common techniques of protein regulation, such as conditional gene expression, RNA interference, knock in, and knock out, partly meet this demand, however, often do not provide sufficient spatial or temporal precision^1^. Antibody-like recombinant binders functional within mammalian cells, generally termed as intrabodies (iBs), were shown to be a powerful technology aimed to regulate protein activity. Small (13–25 kDa)^2^ and highly affine (from nM to low µM)^3^ iBs can be used for allosteric protein regulation, manipulating protein functionality, and for target degradation^4^. Constructs exploiting recombinant binders were used for T-cell-receptor-like signaling reconstruction in nonimmune cells^5^ and for reprogramming of cells into pluripotent stem cells^6^, demonstrating iBs performance in complex synthetic biology applications. More than 3 × 10^6^ antibodies covering about 90% of human proteome^7^, and about 1.5 × 10^3^ single-domain antibodies^8^, potentially used as iBs, are currently reported. Several approaches for selection of specific iBs from libraries^9^ and for their rational design^10^ should make the binders available to almost any cellular protein.

A possibility to switch iBs on and off should advance their functionality. In active (on) state, the knock-out^11–13^ function can be achieved with minimal disturbance, at specific environment and time point. Inactive (off) state can help to avoid off-target influence^14^ by restricting the spatiotemporal binder activity to reduce unwanted interactions.

Natural photoreceptors provided a variety of approaches to design molecular switches, enabling control of protein functions by light^15^. Among these, bacterial phytochromes, sensitive to near-infrared (NIR) light, cryptochromes, and LOV domains, sensitive to blue light, do not need supply of exogenous cofactors to efficiently function in mammalian cells. The distinct spectral sensitivity of these photoreceptors allows their effective spectral multiplexing^16^. The light-dependent structural reorganization, homo- and heterodimerization of these non-opsin photoreceptors were successfully applied to gene expression, regulation of its epigenetic state, control of cell signaling, cell cycle progression, and apoptosis. Blue-light-mediated genome editing was reported recently^17^. The non-opsin optogenetic tools are used in cultured cells of various origin^18^, in primary cell cultures^19^ and in vivo^20^. Although the optogenetic systems to regulate protein–protein interactions demonstrated high spatiotemporal precision and large dynamic range of responces^21,22^, they usually control functions of the overexpressed exogenous proteins. The protein overexpression may result in nonphysiological response, abnormal localization, or aggregation^23^, and suffer from competition with endogenous protein, requiring knocking out the latter by siRNA^24^.

Here, to control native cellular physiology, we integrate different types of iBs, such as nanobodies and monobodies, into NIR and blue-light-activatable optogenetic tools. We thoroughly test compatibility of iBs and optogenetic tools in mammalian cells. We next apply the optogenetic tools merged with iBs to regulate genomically encoded proteins. We show the versatility of this approach by the use of two spectrally resolved optogenetic tools for tridirectional targeting of an endogenous protein. Next, the dual-wavelength optogenetic system for protein localization is applied to regulate cell motility and nuclear actin function. Lastly, we combine the light regulation of endogenous enzymatic activity at one wavelength with a readout of the downstream signaling at another one using spectrally compatible biosensors.

## Results

### Combining iBs with optogenetic system

**Fig. 1:**
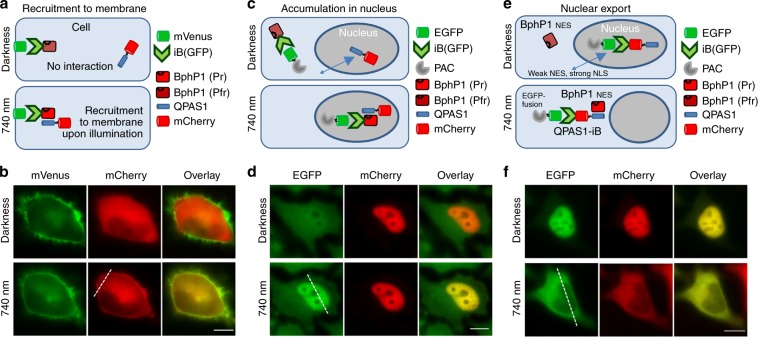
Engineering intrabodies to enable their optogenetic control. **a** Schematic representation of light-induced recruitment of QPAS1-mCherry to the target protein bound to membrane, where interaction with membrane-bound mVenus occurs via intrabody (iB) fused to BphP1. **b** Relocalization of QPAS1-mCherry to plasma membrane under 740 nm illumination. Epifluorescence microscopy; scale bar, 10 µm. **c** Schematic representation of genomically expressed EGFP-PAC relocalization from cytoplasm to the cell nucleus upon illumination. **d** EGFP-PAC relocalization in cells expressing BphP1-iB(GFP) and NES-mCherry-QPAS1-NLS. In darkness, the QPAS1 fusion is shuttling between nucleus and cytoplasm, driven by strong NLS and weak NES. Upon 740 nm illumination, it interacts with BphP1 and recruits EGFP-PAC into the nucleus. Epifluorescence microscopy; scale bar, 10 µm. **e** Schematic representation of nucleus-to-cytoplasm relocalization of genomically expressed GFP-fusion using NIR light-controlled intrabody. **f** Cells expressing genomically EGFP-PAC and transiently BphP1-NES and iB(GFP)-NES-mCherry-QPAS1-NLS. Under 740 nm illumination, EGFP-PAC accumulates in the cytoplasm. Epifluorescence microscopy; scale bar, 10 μm. Fluorescence intensity profiles corresponding the dashed lines in **b**, **d**, and **f** are shown in Supplementary Fig. 1.

To design a light-sensitive construct with iB we fused a bacterial phytochrome from *Rhodopseudomonas palustris* BphP1 (ref. ^25^) to anti-GFP iB vhhGFP4 (ref. ^26^), hereafter referred as iB(GFP). iB(GFP) binds with high affinity *A. victoria*’*s* GFP-derived fluorescent proteins, but not mCherry. BphP1 is a light-sensing component of the heterodimerization optogenetic system consisting of the BphP1 and QPAS1 interacting proteins. Upon absorbing 740–780 nm light, BphP1 undergoes photoconversion into an activated state, resulting in the binding of QPAS1. We monitored this interaction in HeLa cells co-expressing BphP1-iB(GFP), mVenus-CAAX, and mCherry-QPAS1. In darkness, mCherry-QPAS1 localized in cytoplasm. Under NIR light of 740 nm, the mCherry-QPAS1 relocalized to plasma membrane (Fig. 1a, b, Supplementary Figs. 1a and 2). This showed the possibility of light-triggered recruitment of a protein of interest to certain subcellular location using its specific interaction with a recombinant binder. To further characterize this interaction, we studied the kinetics of mCherry-QPAS1 relocalization. The fluorescence signal in cytoplasm decreased with a half-time of 33.6 s (Supplementary Fig. 1b), which was similar to that for interaction of non-fused membrane-targeted BphP1 and QPAS1 (ref. ^27^).

### Targeting genomically encoded protein with iB

Since the expression level of interacting proteins may affect the binding efficiency and kinetics, we further tested iB performance in light-triggered targeting of a genomically encoded EGFP-tagged protein. For this, we established a preclonal mixture of HeLa cells stably expressing EGFP-tagged puromycin N-acetyltransferase (EGFP-PAC) and cotransfected them with BphP1-iB(GFP) and NES-mCherry-QPAS1-NLS. The NES and NLS signals were added to mCherry-QPAS1 to facilitate its shuttling between nucleus and cytoplasm, with the equilibrium shifted to the nucleus, similarly to described^28^ (Fig. 1c, d). In darkness, EGFP-PAC was distributed evenly in nucleus and cytoplasm, being bound to BphP1-iB(GFP). Under 740 nm illumination, BphP1-iB(GFP) interacted with mCherry-QPAS1, resulting in substantial increase of EGFP-PAC in the nucleus, driven by strong NLS sequence of mCherry-QPAS1 (Fig. 1c, d, Supplementary Figs. 1c and 3). Kinetics of this process was slower (*t*_1/2_ = 1890 s) than that for the relocalization from the cytoplasm to the plasma membrane (Supplementary Figs. 1d and 3), likely reflecting its dependence on the nuclear transport machinery and, consequently, on the size of the optically controlled construct itself (in this case BphP1-iB(GFP), ~95 kDa) and the cargo protein (EGFP-PAC, ~48 kDa).

To test a possibility to light-control the opposite process, such as a release of a protein caged in nucleus to cytoplasm, iB(GFP) was fused to mCherry-QPAS1 and the NLS and NES sequences were added for nuclear-cytoplasmic shuttling, shifted to the nucleus in darkness. NES-tagged BphP1 was used to change the localization of iB(GFP)-mCherry-QPAS1 under NIR light (Fig. 1e, f). In darkness, iB(GFP)-mCherry-QPAS1 was predominantly localized in the nucleus, whereas under 740 nm light, due to interaction with strongly cytoplasmic NES-BphP1, both iB(GFP)-mCherry-QPAS1 and EGFP-PAC relocalized to cytoplasm with a half-time of 504 s (Supplementary Fig. 1e, f).

These results show the possibility to light-control subcellular localization of a genomically expressed protein, which can be used, for example, for optical regulation of knocked-in proteins in cell lines generated using CRISPR-Cas9 system^29,30^.

### Three-directional targeting of endogenous proteins

**Fig. 2:**
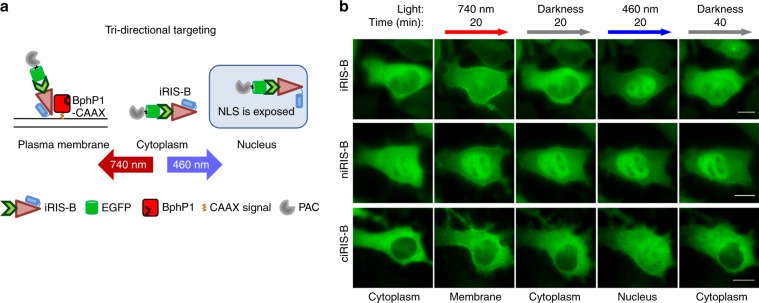
Optogenetic system for three-directional targeting of endogenous proteins. **a** Mode of function of an optogenetic system for the light-controlled tridirectional targeting of endogenous proteins, iRIS-B. **b** Sequential relocalization of EGFP-PAC via iRIS-B (upper), niRIS-B (middle), and ciRIS-B (lower) after irradiation with light of indicated wavelength and recovery due to thermal relaxation. Epifluorescence microscopy; scale bar, 10 μm. Kinetics of EGFP-PAC depletion in cytoplasm, or accumulation in the nucleus via iRIS-B, niRIS-B and ciRIS-B are shown in Supplementary Fig. 4.

Combining optogenetic tools controlled by light of different wavelengths enabled tridirectional protein targeting^27^, which may be used to control multifunctional proteins or to increase dynamic range of bidirectional localizers, as discussed^31^. A NIR-blue-light inducible shuttle (iRIS) construct was shown to be effective for dual-wavelength-controlled exogenous protein relocalization^16,19^. In this system, the QPAS1 protein is fused to a blue-light-sensing AsLOV2-based nuclear localization controller. AsLOV2 carries NLS, which is caged in darkness but becomes accessible after 460 nm illumination, leading to the iRIS accumulation in the nucleus. Under 740 nm light, QPAS1 is interacting with the plasma membrane-anchored BphP1 protein, driving the iRIS to the membrane. Under “drive” or “targeting” we mean the major shift in the equilibrium toward certain compartment, taking into account that the protein of interest is always observed in all compartments but at considerably different concentrations. Here, for tridirectional targeting of genomically expressed proteins, we combined the iRIS with iB(GFP) to make a NES-iB(GFP)-mCherry-QPAS1-AsLOV2cNLS system, termed iRIS-B (Fig. 2, Supplementary Fig. 4, Supplementary Note 1).

**Fig. 3:**
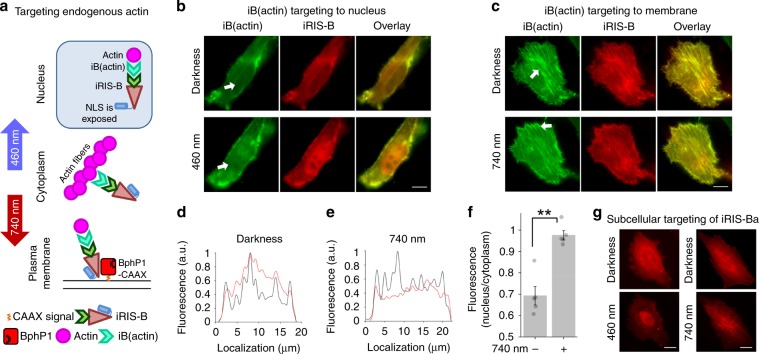
Spectral multiplexing allows targeting of both cytoplasmic and nuclear actin. **a** Schematic representation of dual-wavelength light-controlled perturbation of endogenous non-tagged actin by iB(actin)–iRIS-B system. **b** Optical control of iB(actin) localization in cells transfected with iRIS-B. In darkness, intrabody highlights cortical actin, stress fibers, and is excluded from the nucleus. Under blue light, iB(actin) concentration is increased in nucleus. Epifluorescence microscopy; scale bar, 10 μm. **c** In cells analogous to those shown in **b**, under NIR light, iB(actin) disengages from stress fibers and cytoplasmic structures and binds plasma membrane due to interaction with BphP1-CAAX. Epifluorescence microscopy; scale bar, 10 μm. Intensity profiles of fluorescence signals in cell expressing iB(actin), black line, and iRIS-B, red line, before (**d**) and after (**e**) 740 nm illumination. Respective epifluorescence microscopy image is provided in Supplementary Fig. 6b. Source data are provided as a Source Data file. **f** iB(actin) accumulation in nucleus under 460 nm illumination. Ratios averaged from five regions of interest from image shown in Supplementary Fig. 6a. Error bars represent SEM, **p < 0.01 (two tailed, Student’s *t* test). Source data are provided as a Source Data file. **g** Cells transfected with iRIS-Ba construct in which the system for tridirectional protein targeting (iRIS) is fused to iB(actin) directly. Under blue light, iRIS-Ba concentration is increased in the nucleus; under NIR light, iRIS-Ba is observed at plasma membrane due to its interaction with BphP1-CAAX, similar to iRIS-B. Epifluorescence microscopy; scale bar, 10 μm.

β-actin was reported to be involved in a variety of processes linked to different cellular compartments^32^. Being responsible for vast number of protein–protein interactions, actin is especially sensitive to invasiveness of the approach used to detect or control it. Overexpression of GFP-tagged actin was shown to rise artefacts due to disturbance of normal actin arrangement^33^. For accurate perturbation of endogenous β-actin, we used GFP-labeled camelid iB (iB(actin))^34^ that minimally interferes with normal actin dynamics^33^. To target both nuclear and cytoplasmic actin functions, we apply the iRIS-B system to drive iB(actin) and, subsequently, to cause actin relocalization (Fig. 3a). We cotransfected HeLa cells with iRIS-B and iB(actin), and followed actin arrangement in darkness, under 460 and 740 nm light. In darkness, mCherry signal of iRIS-B and GFP signal from iB(actin) were colocalized with cortical actin and stress fibers (Fig. 3b–d, Supplementary Figs. 5 and 6), with the signal excluded from nucleus. After 460 nm illumination, amount of iB(actin) in the nucleus notably increased (Fig. 3b, f, Supplementary Fig. 6a), nucleoli become visible being highlighted by the signal in nucleoplasm. In turn, after 740 nm illumination, green signal disappeared from stress fibers, concentrating predominantly at the plasma membrane (Fig. 3c–e, Supplementary Figs. 5 and 6b).

**Fig. 4:**
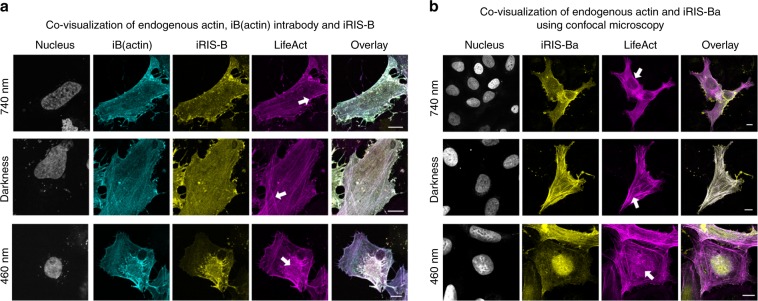
Covisualization of optically controlled iB(actin) and its endogenous target. **a** Colocalization of the components of light-driven iB(actin)–iRIS-B system and light-independent probe for actin visualization (LifeAct-miRFP703 fusion) on plasma membrane (740 nm), on stress fibers and in cytoplasm (darkness), and in the nucleus (460 nm). Arrows indicate compartments highlighted by all three probes. Confocal microscopy; scale bar, 10 μm. **b** Colocalization of iRIS-Ba and light-independent probe for actin visualization (LifeAct-miRFP703 fusion) on plasma membrane (under 740 nm), on stress fibers and in cytoplasm (in darkness), and in the nucleus (under 460 nm). Arrows indicate regions highlighted by both iRIS-Ba and LifeAct. Confocal microscopy; scale bar, 10 μm.

To ensure the targeting of endogenous actin bound to iB(actin), we introduced an additional light-independent probe for actin visualization. For this, we cotransfected cells with iB(actin), iRIS-B, and LifeAct-miRFP703. The cells illuminated with 740 nm, 460 nm, or kept in darkness were fixed and stained with DAPI. The fluorescence signals were distributed (Fig. 4a) in accordance to the scheme in Fig. 3a, with LifeAct-miRFP703 colocalized with iB(actin), confirming the relocalization of endogenous β-actin. The background signal at stress fibers suggests that mainly the monomeric actin pool was affected by relocalization.

To make certain that the iRIS-B–iB(GFP) complex enables the same functionality as a direct iRIS-iB(actin) fusion, we swapped iB(GFP) for iB(actin) in the iRIS-B. The resulting construct was designated as an iRIS-Ba where “a” stands for “actin.” We then imaged iRIS-Ba in live cells under blue and NIR light (Fig. 3g) and in fixed cells in which β-actin was visualized with LifeAct as a light-independent probe (Fig. 4b). In both cases the iRIS-Ba showed the same phenotype as the iRIS-B–iB(actin).

This demonstrated the versatility of iRIS-B, since iRIS-B can be combined with other fluorescently-tagged iBs, enabling the functionality comparable to that achieved by direct fusion. In this way, the approach can be extended to target other endogenous proteins.

### Optical regulation of nuclear actin functions

To study whether actin targeting to nucleus has functional implications, we examined subcellular localization of myocardin-related transcription factor A (MRTF-A), which is a transcription cofactor of serum response factor. Binding of monomeric actin to the RPEL-domain of MRTF-A regulates nucleocytoplasmic shuttling of MRTF-A by inhibiting its nuclear import and enhancing nuclear export^35^.

**Fig. 5:**
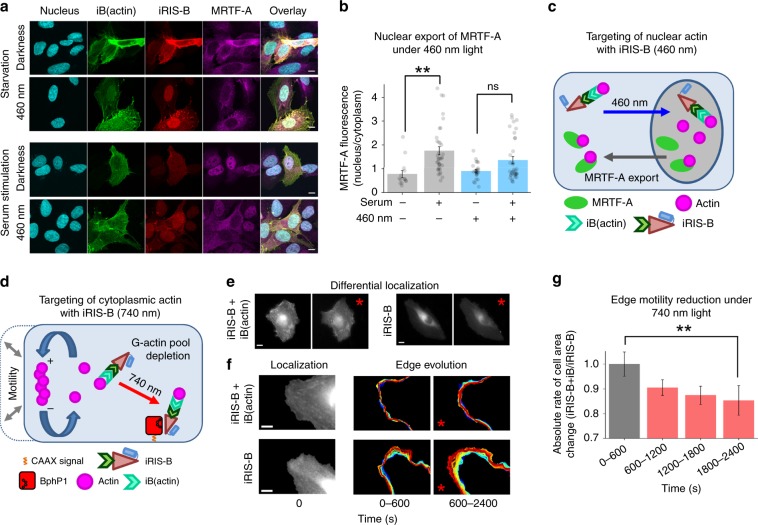
Light-controlled regulation of cell motility and nuclear actin functions. **a** Light-dependent nuclear export of MRTF-A in cells co-expressing iRIS-B and iB(actin). After cell fixation, MRTF-A was visualized using immunostaining. Confocal microscopy; scale bar, 10 µm. **b** MRTF-A localization in cells expressing iB(actin)–iRIS-B system, error bars represent SEM, *n* = 14–35 imaged cells. **p < 0.01, ns nonsignificant at *p* = 0.01 (two tailed, Student’s *t* test). Source data are provided as a Source Data file. **c** Schematic representation of light-regulation of non-tagged nuclear actin by iB(actin)–iRIS-B system. **d** Schematic representation of light-control of cytoplasmic actin by iB(actin)–iRIS-B system. **e** NIR light-triggered perturbation of endogenous cytoplasmic actin by iRIS-B and iB(actin). 740 nm illumination is indicated by red asterisk. Epifluorescence microscopy; scale bars, 20 µm. **f** Zoom-in views of the live-cell imaging of NIR light-triggered perturbation of endogenous cytoplasmic actin, as in **e**. Red asterisk indicates illumination by 740 nm light. Epifluorescence microscopy; scale bars 10 µm. **g** Absolute values of the rate of cell area change during time-lapse experiments. Optogenetic sequestration of endogenous actin to the plasma membrane significantly impacted cell edge motion dynamics as a function of time. Data are shown as the ratio of average results from the condition in which both iB(actin) and iRIS-B were co-expressed, from the negative control condition in which only the iRIS-B was expressed. Light-activation started at 600 s time point and remained for the entire duration of assays thereafter. Representative examples of edge tracking traces are shown in Supplementary Fig. 8. *n* = 29–35 protrusion/retractions pooled from 3–4 independent experiments, shown with SEM, **p < 0.01 (two tailed, Student’s *t* test). Source data are provided as a Source Data file.

We illuminated serum-starved U2OS cells co-expressing the iRIS-B and iB(actin) with 460 nm light to enrich actin in nucleus, as in (Figs. 3b, f and 4a). The cells were then stimulated with serum, fixed, and MRTF-A subcellular localization was examined by immunostaining. In the serum-starved conditions (Fig. 5a, upper panel), MRTF-A was mainly cytoplasmic, as reported earlier;^35^ and the 460 nm illumination almost did not affect its localization. However, in the serum-stimulated conditions (Fig. 5a, lower panel, Supplementary Fig. **7**), MRTF-A was less nuclear in cells illuminated with 460 nm light than in cells kept in darkness. In agreement with previous studies showing that the actin binding to MRTF-A is required for its nuclear export^35^, the increased nuclear actin stimulated the nuclear export of MRTF-A (Fig. 5b, c).

These experiments further demonstrated that the iRIS-B system can be efficiently used to study compartment-specific functions of endogenous β-actin.

### Optical control of cell motility via endogenous actin

An ability to regulate endogenous cytoplasmic β-actin could provide a new approach to perturb cell motility (Fig. 5d). We cotransfected HeLa cells with iB(actin) and iRIS-B and imaged random cell motion in serum for 2400 s (Fig. 5e, f). In darkness, mCherry-iRIS-B localized similar to the F-actin distribution visualized through the iB(actin) GFP channel (Fig. 5e, Supplementary Movie 1). Upon 740 nm illumination, started at 600 s of cell imaging, the cellular distribution of mCherry-iRIS-B shifted from the F-actin-like to a punctuated distribution that is reminiscent of the plasma membrane localization (Fig. 5e, Supplementary Movie 1). This was corroborated by control experiments in which the iB(actin) was omitted from co-transfection, but cells were optogenetically activated to undergo the cytoplasm-to-plasma membrane transition of iRIS-B (Fig. 5e, Supplementary Movie 2). We quantified the absolute value of the rate of cell area change during random motility driven by serum and with 740 nm optogenetic control of endogenous actin compared with the control without targeting of actin (Fig. 5f, g). The results indicated an immediate reduction in the ability of cells to move the edge efficiently (both in protrusion and in retraction) after switching on 740 nm light (600–2400 s), which reached a maximum and highly significant attenuation (*p* < 0.01 (two tailed, Student’s *t* test)) by 1800–2400 s (Fig. 5f, g). Changes in the edge motion dynamics is visualized and quantified by computationally tracking the edge (Supplementary Fig. 8, Supplementary Movies 3 and 4), resulting in visible differences in the motility dynamics of cells when 740 nm is irradiated together with co-expressions of iRIS-B and iB(actin).

These results show that the light-controlled perturbation of endogenous cytoplasmic actin by the orthogonal optogenetic tool impacts the protrusive dynamics and cell motility.

### Engineering of optogenetic control of endogenous GTPase

To study an ability to light-control of cellular endogenous enzymes, we next designed NIR-sensitive constructs combining an iB and a BphP1-QPAS1 optogenetic system for nucleus-to-cytoplasm relocalization. We chose an NS1 monobody specific to RAS GTPase^36^ (hereafter iB(RAS)), a well-characterized membrane-bound oncogenic protein. iB(RAS) was shown to allosterically regulate activity of the H- and K-RAS isoforms.

**Fig. 6:**
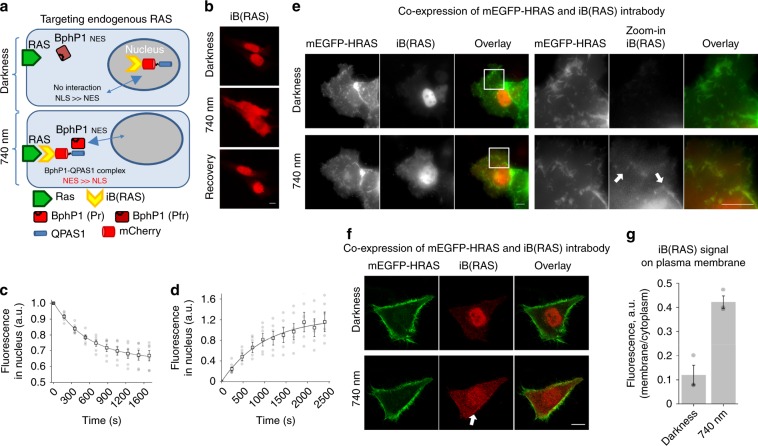
Engineering of light-controlled intrabody for endogenous RAS. **a** Schematic representation of a regulation of the intrabody against RAS, designated “iB(RAS),” by NIR light. In darkness, the intrabody shuttles between nucleus and cytoplasm, being predominantly in the nucleus. Under 740 nm light, the iB(RAS)-QPAS1 fusion interacts with BphP1-NES and accumulates in the cytoplasm, due to the summation of NES signals from both interacting partners. As a result, the intrabody can reach endogenous RAS on the plasma membrane. **b** Cells co-expressing BphP1-NES and iB(RAS)-NES-mCherry-QPAS1-NLS. mCherry fluorescence is observed in the nucleus in darkness and in the cytoplasm under 740 nm light. The process is reversible: in darkness the iB(RAS) moves back to the nucleus. Epifluorescence microscopy; scale bars, 10 µm. **c** Kinetics of iB(RAS) depletion in the nucleus as detected by mCherry fluorescence, error bars represent SEM, *n* = 6 cells. Source data are provided as a Source Data file. **d** Kinetics of the light-driven iB(RAS) accumulation in nucleus (recovery) as a result of BphP1 thermal relaxation, error bars represent SEM, *n* = 7 cells. Source data are provided as a Source Data file. **e** HeLa cells co-expressing the optically controlled iB(RAS) (red) and mEGFP-HRAS (green). Zoom ins of white frame-marked regions are shown on the right. White arrows show the membrane structures where iB(RAS) and mEGFP-HRAS co-localize. Epifluorescence microscopy; maximal projection is shown; scale bar, 10 µm. **f** Relocalization from nucleus to cytoplasm of optically controlled iB(RAS) (red) under 740 nm illumination, with subsequent binding of membrane-associated mEGFP-HRAS (green), white arrow. Confocal microscopy; scale bar, 10 µm. **g** Mean fluorescence signal on plasma membrane, shown as membrane-to-cytoplasm ratio, quantified for three cells similar to shown in **f**. Error bars represent SEM. The scheme used for the quantification is presented in Supplementary Fig. 9. Source data are provided as a Source Data file.

iB(RAS), which was fused to mCherry-QPAS1 and tagged with strong NLS and weak NES tags for nucleocytoplasmic shuttling, had localization substantially shifted to the nucleus. iB(RAS)-mCherry-QPAS1 was further cloned after NES-tagged BphP1 via T2A self-cleavable peptide. In this way, BphP1 activated by NIR light caused a traction that shifted the nucleus-to-cytoplasm equilibrium of iB(RAS) toward the cytoplasm (Fig. 6a). Testing of the resulting construct showed the accumulation of iB(RAS) in the cytoplasm with a half-time of 516 s and its recovery in darkness with a half-time of 690 s (Fig. 6b–d, Supplementary Note 2). Next, we studied if optically controlled iB(RAS) could recognize its target. For this, mEGFP-HRAS fusion was co-expressed with iB(RAS). Similar to the initial tests, iB(RAS) accumulated in the nucleus in darkness. Under 740 nm light, it was released to the cytoplasm and highlighted the membrane structures, being colocalized with mEGFP-HRAS (Fig. 6e–g, Supplementary Fig. 9). This demonstrated that the binding properties of anti-RAS iB were not affected due to integration into the light-sensitive system. Importantly, we observed a very low background signal from iB(RAS) in the cytoplasm before NIR illumination.

**Fig. 7:**
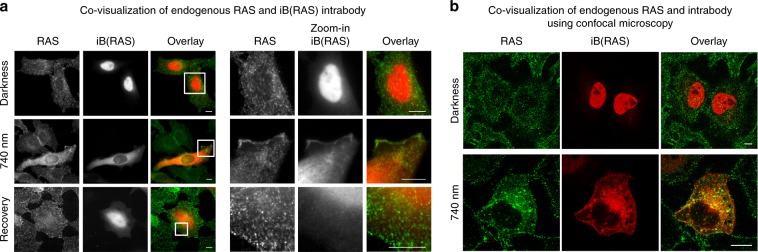
Covisualization of optically controlled iB(actin) and its endogenous target. **a** HeLa cells expressing the optically controlled iB(RAS) (shown in red) were stained with anti-pan-RAS monoclonal antibodies (green). iB(RAS) has nuclear localization in darkness. Under 740 nm light, it is released from nucleus to cytoplasm and plasma membrane where it is colocalized with endogenous RAS. Zoom in of white frame-marked regions are shown on the right. Epifluorescence microscopy; scale bar, 10 µm. **b** Cells with the NIR light-triggered release of iB(RAS) imaged using confocal microscopy. Scale bar, 10 µm.

Next, the optically controlled iB(RAS) was imaged with its endogenous target. For this, cells kept in darkness, after 1800 s of 740 nm illumination, and after 1800 s of 740 nm illumination followed by 1800 s of darkness, were fixed and stained with anti-pan-RAS monoclonal antibody (mAb). Endogenous RAS was observed predominantly near the cell perimeter (Fig. 7a, Supplementary Fig. 10), with high concentration in the cell ruffles, which was in accordance with the published data^37,38^. iB(RAS)-mCherry-QPAS1 under illumination showed mostly even cytoplasmic distribution, with slightly higher signal in cell ruffles where anti-RAS mAbs were concentrated (Fig. 7a). Under NIR light, cells with uncaged iB(RAS) showed less RAS at the plasma membrane than nontransfected cells. These results were in accordance with the original iB(RAS) paper^36^ and abundance of RAS isoforms in HeLa cells^39^. Importantly, simultaneous visualization of iB(RAS)-mCherry-QPAS1 with endogenous RAS stained with mAbs allowed to exclude their colocalization in darkness. Under NIR illumination, iB(RAS)-mCherry-QPAS1 concentration in cytoplasm increased, enabling access to RAS. To additionally verify the interaction between endogenous RAS and iB(RAS), we imaged the same samples using confocal microscopy. We observed the similar distribution pattern of RAS (visualized by immunostaining) and iB(RAS) after its release to the cytoplasm under 740 nm light (Fig. 7b). After dissociation of the BphP1-QPAS1 complex during dark relaxation (bottom row in Fig. 7a) and subsequent iB(RAS)-mCherry-QPAS1 depletion from cytoplasm, the RAS and iB(RAS) interaction seems not likely.

The distinct iB(RAS)-mCherry-QPAS1 and RAS distributions (even cytoplasmic versus dotted, respectively) were likely caused by different affinities of iB(RAS) and mAbs to different RAS isoforms and possible competitive binding of iB(RAS) and mAbs.

### Optical regulation of endogenous GTPase signaling

We next studied whether cell signaling can be perturbed via allosteric inhibition of endogenous RAS by optically controlled iB(RAS). The RAS-initiated mitogen activated protein kinase (MAPK) pathway culminates in transcriptional regulation by activated extracellular-signal regulated kinase (ERK)^40,41^. Subcellular localization of ERK depends on phosphorylation, therefore, fluorescently labeled ERK localization is used as a readout of MAPK pathway activity^42^. We hypothesized that light-driven inhibition of RAS by iB(RAS) should influence the MAPK signaling in serum-starved HeLa cells under acute stimulation by epidermal growth factor (EGF).

**Fig. 8:**
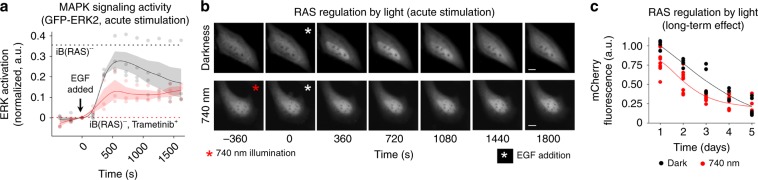
Endogenous RAS regulation using light-controlled intrabody. **a** Light regulation of MAPK pathway in cells co-expressing optogenetically controlled iB(RAS) and GFP-ERK2. Signal in cells preilluminated with 740 nm light for 20 min before *t* = 0 s is shown in red, signal in cells kept in darkness is in gray. Filled areas represent SEM, *n* ≥ 3. Dashed lines show ERK activation level at *t* = 540 s, in cells expressing only GFP-ERK2 reporter, in presence of 500 nM trametinib (mean = 0.001, shown in red; SEM = 0.023) or without inhibitor (mean = 0.355, shown in black; SEM = 0.052). Source data are provided as a Source Data file. **b** EGF stimulation of cells expressing iB(RAS) and GFP-ERK2, in darkness and under 740 nm illumination. Epifluorescence microscopy; scale bar, 10 µm. **c** Cells expressing system for RAS light-regulation show lower survival rate under 740 nm (red line) as compared with those kept in darkness (black). Source data are provided as a Source Data file.

To test this, HeLa cells co-expressing light-controlled iB(RAS) and GFP-ERK2 were stimulated with EGF either in darkness or after 1200 s of NIR illumination. Cells expressing only GFP-ERK2 reporter, but not optogenetic controller (designated as iB(RAS)^–^), were used for the estimation of the system dynamic range. For this, ERK activation level was probed in darkness, after EGF stimulation, in presence or absence of 500 nM trametinib, which is potent inhibitor of MEK^43^. In optogenetically regulated cells, in darkness, EGF triggered the prompt relocalization of GFP-ERK2 to the nucleus, with peak activation within first ~720 s of stimulation (Fig. 8a, b), as previously described^43^. After this, MAPK activity slowly decreased. In contrast, in preilluminated cells, EGF-stimulated ERK activity was substantially lower, reaching a plateau at twice-lower level as compared with cells in darkness, and remaining at this level for at least 1000 s (Fig. 8a, b). Importantly, the EGF activation level in iB(RAS)^–^ cells was close to that of the iB(RAS)-expressing cells in darkness, showing the effectiveness of iB caging in the nucleus. We concluded that optogenetic control of iB(RAS) enables regulation of MAPK signaling.

To probe a long-term effect from RAS signaling inhibition, we monitored viability of HeLa cells expressing iB(RAS)-mCherry-QPAS1 in darkness and under NIR light using flow cytometry. RAS inhibition is a well-known trigger of cell death in various cancer cell lines, including HeLa^44^. Indeed, the long-term 740 nm illumination decreased the survival rate of cells expressing iB(RAS), likely due to the RAS inhibition (Fig. 8c), additionally proving the functional activity of optogenetically controlled iB(RAS).

### All-optical control of endogenous enzymatic activity

To further characterize the optogenetic modulation of endogenous RAS by iB(RAS), we chose a biosensor for ERK activity, EKAR2G2, to monitor ERK-mediated phosphorylation downstream of RAS signaling subnetwork. EKAR2G2 is based on the Förster resonance energy transfer (FRET) between cyan and yellow fluorescent proteins^45^ and reports the local cellular equilibrium of phosphorylation activity by ERK and dephosphorylation activity by cellular phosphatases. By monitoring the FRET/donor ratio from EKAR2G2 one may infer the ability of ERK to phosphorylate its target substrate^45^.

**Fig. 9:**
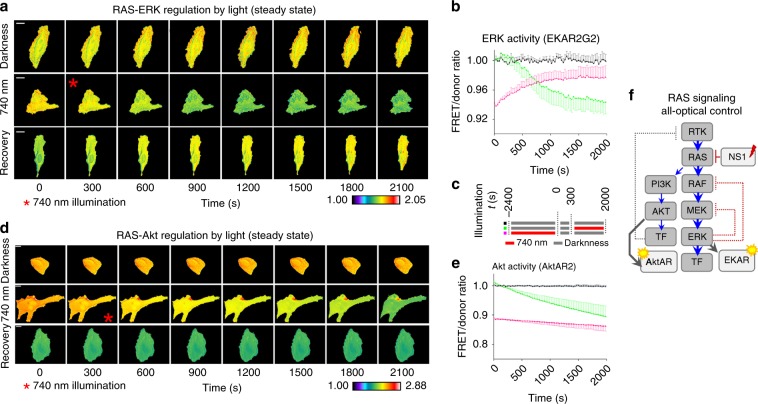
All-optical control of endogenous RAS in steady state. **a** RAS regulation by light in cells expressing EKAR2G2, in serum. Epifluorescence microscopy; scale bar, 20 µm. Pseudocolor scale: black = 1.0 and white = 2.05. **b** Normalized whole-cell average FRET/donor ratio in HeLa cells expressing the optogenetically enhanced iB(RAS) and EKAR2G2 FRET biosensor. *n* = 3, error bars represent SEM. Source data are provided as a Source Data file. **c** Illumination scheme used for the experiments shown in Fig. 9. **d** RAS-Akt signaling regulation in cells expressing optogenetically controlled iB(RAS). Representative time-lapse panels of AktAR2 FRET/donor ratio, imaged in HeLa cells in serum. Epifluorescence microscopy; scale bar, 20 µm. Pseudocolor scale: black = 1.0; white = 2.88. **e** Normalized whole-cell average FRET/donor ratio as a function of time, in HeLa cells expressing the AktAR2 FRET biosensor. Error bars represent SEM, *n* = 3 independent experiments. Source data are provided as a Source Data file. **b**, **e** Black: control cells without light-activation. Green: cells with 740 nm illumination starting at *t* = 300 s time point. Magenta: cells were irradiated with 740 nm light for 2400 s prior to imaging. **f** Schematic representation of all-optical control of RAS signaling using iB(RAS). Signaling nodes are shown in gray, optogenetic tools and biosensors are shown in light gray. Fast negative feedback loops acting via phosphorylation are shown as red dotted lines. Slow feedback loops acting via transcription inhibition are shown in gray.

First, we chose to determine an effect of optogenetic release of RAS-bound iB(RAS) in HeLa cells in serum under steady-state conditions (Fig. 9a–c, Supplementary Figs. 11 and 12, Supplementary Movies 5–7). Upon 740 nm irradiation, the FRET/donor ratio of EKAR2G2 decreased rapidly as a function of time (*t*_1/2_ ~450 s) to reach a plateau (Fig. 9b). The reduction of FRET/donor ratio indicated a relative loss of the ability of ERK to phosphorylate its substrate when the iB(RAS) was released from the nuclear sequestration. Conversely, when cells were irradiated at 740 nm for 2400 s prior to the start of EKAR2G2 imaging (740 nm light was then switched off), the ability of ERK to phosphorylate its substrate partially recovered and approached an elevated plateau during the dark relaxation (Fig. 9a, b). The rate of recovery during the dark relaxation (half-time of 750 s) was slower as compared with the rate of inhibition from the light-induced release. The extent of recovery also did not reach the full steady-state levels, suggesting either an inefficient re-shuttling of iB(RAS) into the nucleus or that activated ERK may negatively feedback to attenuate the pathway at RAF/MEK while attaining homeostatic equilibrium^41^ (Fig. 9f). We also determined the total dynamic range of ERKAR2G2 under steady-state conditions using MEK inhibitor trametinib (500 nM), which led to RAS-ERK downregulation comparable to those triggered by optogenetics (Supplementary Fig. 12), confirming efficient performance of iB(RAS).

Next, we followed an activity of another RAS-driven signaling subnetwork, RAS-Akt, using an AktAR2 FRET biosensor. In contrast to the RAS-ERK, the RAS-Akt signaling did not show significant recovery in darkness after downregulation by optically controlled iB(RAS) (Fig. 9d, e). This difference is likely originated from mechanisms underlying RAS-ERK and RAS-Akt regulation, as the former is regulated mostly through phosphorylation, whereas the latter may be controlled primarily by transcription (Fig. 9f, Supplementary Notes 3 and 4).

These results showed that endogenous RAS signaling can be optogenetically regulated using NIR light and simultaneously monitored using biosensors for downstream targets operating in visible-light spectral range, enabling an all-optical assay approach. This allows functional characterization of the signaling subnetworks based within their feedback timescales, imperative for direct interrogation of cellular metabolism^46,47^.

## Discussion

Although recombinant binders of endogenous targets are widely used in research and therapy, the inability to control them with high temporal precision leads to off-target activity and requires invasive delivery approaches^48^. To overcome these obstacles, we, for the first time, incorporated iBs into the BphP1-QPAS1 optogenetic construct, enabling highly specific, fast, and noninvasive functional regulation of endogenous proteins.

Using an overexpressed protein target (Fig. 1a, b), we first validated that strong and specific interaction of an iB with the target suffices to drive the protein relocalization with the kinetics comparable to that of the original optogenetic construct. This prompted us to proceed with the designing of an optogenetic system to control genomically expressed GFP-tagged proteins. We next demonstrated that the concentration and, hence, the function of the protein target in a particular cellular compartment are effectively controlled by light (Fig. 1c–f). Notably, the developed iB(GFP)-incorporating optogenetic system can be applied to any GFP-knocked-in cell line in which an endogenous protein is fused with GFP-like tag, making it the perfect complementary tool for cell lines generated by CRISPR-Cas9 genome editing^29,49^.

This optogenetic approach could be multiplexed in terms of using several iBs of different specificity. Combination of iBs for different targets would be useful for applications in which the iBs being inactive individually could trigger a biological process after the complex formation, as in a case of allosteric inhibition of Abl^50^.

We next used a spectral multiplexing of two distinct light-sensing moieties in iRIS-B (Fig. 2) for dual-wavelength control of endogenous β-actin (Figs. 3–5). iB(actin) that minimally impacts actin dynamics^51^ and iRIS-B were combined to induce measurable effects on cellular motility. Our results indicate significant reduction of overall protrusion–retraction rate under NIR light, supporting the idea that sequestering endogenous actin at the plasma membrane slows down the overall rate of cellular morphodynamics driven by actin cytoskeletal rearrangement. The same iB(actin)-iRIS-B combination illuminated with blue light led to the increase of nuclear actin pool, followed by the MRTF-A nuclear export, demonstrating the possibility to regulate multifunctional proteins using dual-wavelength-controlled optogenetic systems. Future studies with optogenetically enhanced iB(actin) could consist of local actin perturbations in lamellipodia, filopodia, podosomes, and tunneling nanotubes, providing further insights into the role of actin dynamics in these cellular structures.

Spectral multiplexing of the NIR light-controlled system for RAS activity with the visible-light ERK and Akt protein kinase biosensors^45^ enabled an all-optical assay of endogenous RAS downstream signaling (Figs. 8 and 9). Monitoring of two distinct subnetworks of RAS signaling showed the innate difference in their regulation mechanisms. Likely, the observed dissimilarity in the recovery kinetics of the RAS-ERK and RAS-Akt subnetworks suggests a fast feedback control of RAS-ERK by intermediate kinases, whereas RAS-Akt may be controlled by transcriptional regulation, exhibiting considerably longer feedback timescales (Fig. 9f, Supplementary Note 4). It is also possible that Akt could be directly impacted by other pathways, in addition to RAS, including PDK1, mTORC2, and protein phosphatases. With the ability to extend the iB-incorporating optogenetic approach to any available iB, including for other modulators of Akt activity, the analyses of cell signaling are now made possible in living cells by targeting endogenous, unmodified proteins for direct optical perturbation.

Despite the described optically controlled iBs show the high potential for regulation of endogenous proteins, several factors make troubleshooting necessary in its applications. First, the performance of an iB-based optogenetic tool depends on the characteristics of the used iB. Hence, the mechanism of action, affinity and specificity, being the features of iB, are inherited by the optogenetic tool. It may happen that too strong binding of iB to its target can increase system leakage in darkness. However, this can be potentially resolved by adjusting the iB affinity or by choosing the different iB. Second, the described optogenetic systems have relatively slow kinetics of thermal relaxation (switching off). In future, this can be addressed by directed evolution of light-sensitive proteins into improved versions with the faster off kinetics. Third, although the bacterial phytochrome-based optogenetic tools benefit from the abundance of biliverdin in mammalian cells, they may depend on its concentration in a particular tissue. This technical obstacle can be overcome by using stable expression of the phytochrome part of the iB-incorporating optogenetic tools^16^.

To summarize, we have demonstrated, for the first time, that the optogenetically enhanced iBs allow precise regulation of endogenous proteins otherwise hard-to target with small molecules or genetically encoded probes, which is the case for both RAS small GTPase^14^ and β-actin^33^. Moreover, the developed iB-based light-control technology may help to reduce side effects of therapeutic iBs by limiting their action to a specific subcellular location or a cell type and to a certain time period, avoiding the undesirable influence on healthy cells and tissues.

## Methods

### Design of plasmids

The plasmids designed in this study are summarized in Supplementary Table 1. Mammalian vectors used in this study used plasmids designed by us earlier as templates^16^. Anti-GFP nanobody vhhGFP4, referred in this paper as “anti-GFP iB” or “iB(GFP)” was from pcDNA3_NSlmb-vhhGFP4, a gift from M. Affolter (Addgene plasmid #35579). pQP-2376 was used as an intermediate cloning step, NSImb domain was further removed, to get pQP-2460. Vector containing PiggyBac^52^ terminal repeats and EGFP-PAC under CMV promoter, as well as vector for hyperactive PiggyBac transposase expression were constructed by VectorBuilder (Cyagen Biosciences). pLentiEKAR2G2 was a gift from O. Pertz (Addgene plasmid #40178). pcDNA3-AktAR2 was a gift from J. Zhang (Addgene plasmid #64932). GFP-ERK2 was a gift from R. Seger (Addgene plasmid #37145). mEGFP-HRAS plasmid was a gift from K. Svoboda (Addgene plasmid #18662). Anti-RAS monobody NS1, referred in this paper as “iB(RAS)” was synthetized by GenScript, using amino acid sequence from 5E95 Protein Data Bank entry for reverse translation^53^. Actin chromobody, referred in this paper as “iB(actin)” is commercially available from ChromoTek.

### Mammalian cell culture and transfection

HeLa cells were purchased from the ATCC (CCL-2) and were not additionally authenticated or tested for mycoplasma contamination. Cells were grown in DMEM medium supplemented with 10% FBS, penicillin-streptomycin mixture (all from Gibco) at 37 °C. For experiments, cells were plated on six-well plates (Greiner Bio-One). Transient transfections were performed using an Effectene transfection reagent (Qiagen). The culture medium was changed 6 h after the transfection with the new one containing 25 μM of BV. Preclonal mixtures of HeLa cells were obtained using the plasmid-based PiggyBac transposon system. For this, the EGFP-PAC under CMV promoter was cloned into the transposon bearing plasmids pQP-EGFP-PAC and cotransfected with a plasmid encoding a hyperactive PiggyBac transposase, in 1 (transposon):3 (transposase) ratio. Cells were further selected with 3 µg/ml of puromycin (InvivoGen) for 2 weeks resulting in the preclonal HeLa cell mixtures stably expressing EGFP-PAC.

For EKAR2G2 (Addgene #40178) and AKTAR2 (Addgene #64932) biosensor experiments, HeLa cells were transiently transfected together with pQP-AR10 (Supplementary Table 1) construct at DNA ratio of 1: 1. For iB(actin)–iRIS-B experiments, HeLa cells were transiently transfected with iB(actin), stands for actin chromobody (ChromoTek), and iRIS-B DNA at a ratio of 1:3. Cell transfections were performed by using PEI (ref. ^54^) and Fugene HD (Promega) reagents following the manufacturer’s protocols. HeLa cells were trypsinized and plated onto 6-well dishes at 1.5 × 10^5^ cells per well on the morning of transfection. Two hours after plating, transfection was performed with total DNA of 2 µg/well. Six hours following transfection, media was supplemented with 25 µM BV and incubated overnight. Live-cell imaging experiments were performed starting at 24 h post transfection. Recombinant human EGF (Thermo Fisher, PHG0315) was used for acute stimulation, at final concentration of 25 ng/ml (4 nM).

For nuclear actin regulation studies, 2 days prior to the experiment, human osteosarcoma (U2OS) cells were seeded on coverslips with DMEM supplemented with 10% FBS. Next day, the cells were transfected with iRIS-B and actin chromobody (ChromoTek) 2:1 ratio using JetPrime (Polyplus-Transfection) transfection reagent according to the manufacturer’s protocol. Eight hours after transfection, the media was changed to DMEM with 0.3% FBS and 25 µM of BV to introduce serum starvation. After 16 h in starvation, the cells were either illuminated with 460 nm light (1 mW cm^−2^) or kept in darkness for 30 min. FBS was then added to the media to raise the total FBS concentration to 15% in order to cause serum stimulation. Cells were fixed with 4% paraformaldehyde after 1 min of serum stimulation.

### Cell light activation and imaging

Imaging was performed using an Olympus IX83 inverted epifluorescence microscope equipped with a 200 W metal halide (Lumen 220PRO, Prior) or a xenon-arc (Lambda LS, Sutter) lamps. An OptiMOS sCMOS (QImaging) or an ORCA-Flash4.0 V3 (Hamamatsu) cameras were used for image acquisition. Unless otherwise indicated, cells were imaged using either a 40×, 0.95 NA air or a 60 × 1.35 NA oil objective lens (UPlanSApo, Olympus). HeLa cells imaging was performed in a live-cell imaging solution (Invitrogen, A14291DJ) at 37 °C. CELLview glass‐bottomed dishes (Greiner Bio‐One) were used for imaging. Confocal imaging was performed by using a Leica TCS SP8 microscope equipped with a 63 × 1.4 NA, and HC PL APO CS2 objective and a white light laser (470–670 nm).

For the relocalization assays, NIR or blue (both 1 mW cm^−2^) illumination was applied by using the custom‐assembled LED arrays (LED Engin), 460/20 and 740/25 nm respectively. Focusing of the microscope was performed in mCherry channel to prevent unspecific activation of blue‐ and NIR‐light‐sensing components. A pulsed illumination (10 min 740 nm/30 min darkness) was used for a long-term survival assay.

The data were analyzed using a SlideBook v. 6.0.8 (Intelligent Imaging Innovations), a Fiji v. 1.50b (ref. ^55^) and an OriginPro v. 8.6 (Origin Labs) software. Images were appropriately scaled for meaningful representation. Specifically, a lookup table altering was performed for reduction of background signal; a maximal signal value specification and a nonlinear correction (gamma in the range of 0.8–1) was performed to avoid picture oversaturation, if necessary. Unless indicated to the contrary, a single Z-section is shown. No deconvolution techniques were used. Importantly, for all types of quantification, such as (i) intensity measurements, (ii) profiles plotting, and (iii) colocalization analysis, the uncompressed and unmodified 16-bit TIFF images were used. A Savitzky–Golay denoising (points of window = 60, polynomial order = 2) was applied to intensity profiles using an OriginPro v. 8.6 software (Origin Labs). Time-lapse images were combined in stacks and aligned using a rigidbody registration algorithm. To measure the kinetics of fluorescence intensity, at least eight separate regions of interest in the cytoplasm or in the nucleus were chosen for each cell. The background fluorescence was subtracted from the mean fluorescence intensity for each time point, and the intensity levels were normalized to the initial fluorescence and plotted. Fluorescence intensity profiles were determined using an ImageJ software.

Image analysis for quantification of fluorescence associated with the plasma membrane was performed using the NumPy array operations, morphological operations, filtering from SciPy, and plotting from Matplotlib, all available from an Anaconda python distribution (v. 2019.03).

For MRTF-A relocalization experiments cells were imaged using Leica TSC SP8 confocal microscope (Leica Microsystems) with HC PL APO 93 × 1.3 NA glycerol objective. Confocal sections from the middle of the cells were recorded and the intensity was measured from both the nucleus and the cytoplasm with 40 × 40 pixels circle selection using FIJI. The ratio between the nucleus and the cytoplasm was calculated and average ratios plotted with standard error of the mean (SEM).

### Fixed samples preparation and immunostaining

HeLa cells transfected with pQP-AR10 were cultured on coverslips, coated with poly-L-lysine (Merck, A-005-C) and (i) kept in darkness, (ii) illuminated by 740 nm light (30 min), or (iii) preilluminated by 740 nm light (30 min) with following recovery period (30 min in darkness). After this, cells were fixed with 4% paraformaldehyde for 10 min at room temperature, washed three times with PBS, and permeabilized for 15 min with 0.5% Tween 20. After blocking with 1% BSA in PBS containing 0.1% Tween 20 (PBST, 1 h) cells were incubated with primary Anti-Pan-Ras antibody, mouse monoclonal IgG2aκ, clone RAS 10 (MABS195, Merck), 1:500 dilution, for 1 h, in 1% BSA in PBST. Further, coverslips were washed and incubated with secondary Alexa Fluor 488‐conjugated antibody, anti-mouse goat IgG (Thermo Fisher, A-11001) for 1 h in 1% BSA in PBST, then washed and mounted using mounting medium containing DAPI (Santa Cruz). For nuclear actin regulation studies, fixed cells were stained with MRTF-A antibody (G-8, sc-390324, Santa Cruz Biotechnology), 1:500 dilution, followed by anti-mouse Alexa Fluor 647 secondary antibody (Thermo Fisher) and DAPI.

### Imaging of FRET biosensor and the optogenetic tool

HeLa cells transfected with the biosensor and pQP-AR10 were plated onto 25 mm no. 1.5 coverslips coated with fibronectin (10 µg/ml), under normal culture conditions without BV and in the dark for 3 h prior to imaging. Cells were transferred to imaging medium consisting of Ham’s F-12K without phenol red, supplemented with 3% FBS, argon gas sparged, and treated with Oxyfluor reagent with 5 mM NaC_3_H_5_O_3_ (www.oxyrase.com). FRET imaging was performed following previously described methods^56^. Briefly, coverslips containing cells were mounted onto a temperature regulated sealed chamber system^57^ atop a widefield epifluorescence Olympus IX81-ZDC microscope (Olympus). Cells were imaged through a ×40 magnification objective lens (Olympus UIS 40 × 1.3 N.A.), illuminated with light from a 100 W Hg arc lamp via an excitation filter ET436/20X (Chroma Technology). Fluorescence emission was routed through an external beamsplitter via an T505LPXR (Chroma Technology) that separated the FRET and CFP channels, and captured simultaneously via two independently mounted CoolSnapES2 cameras (Photometrics) through bandpass filters ET480/40M for CFP and ET535/30M for FRET (Chroma Technology). For optogenetic activation, 75 W Xe arc lamp was used to illuminate through a bandpass filter HQ745/45X (Chroma Technology) and was combined with the main fluorescence excitation light train via a long-pass mirror T650LPXXRU (Chroma Technology). The illumination shutter for the optogenetic activation was controlled manually as required during the imaging experiment to irradiate the whole field with the far-red excitation light. Images were acquired at 10 s intervals for the given duration of time per experiment. For data analysis, FRET and donor channel images were camera noise-, background-, and flatfield corrected^56^. The two channels were then processed for proper alignment using a priori calibration and a nonlinear coordinate transformation approach to achieve pixel-to-pixel matching required for ratiometric analysis^56^. Cell images were thresholded based on the intensity histogram to select the foreground region from the background, binary masks were created, and multiplied into the cell images to achieve segmentation of the data. These images were then *x*–*y* translationally aligned^58^, and the FRET channel was divided by the CFP channel to result in ratiometric data. A linear lookup table was applied that corresponded to relative biosensor activity levels within a cell.

### Cell area change quantification

For iB(actin) + iRIS-B analysis, HeLa cells expressing iB(actin) and iRIS-B were plated onto fibronectin (10 µg/ml) coated 25 mm #1.5 coverslips and allowed to attach for 3 h under normal culture conditions (without BV) in the dark prior to imaging. Cells were transferred to imaging medium consisting of Ham’s F-12K without phenol red, supplemented with 10% FBS, argon gas sparged, and treated with Oxyfluor reagent with 5 mM NaC_3_H_5_O_3_ (www.oxyrase.com). Coverslips containing cells were mounted onto a temperature regulated sealed chamber system, and imaged at 10 s intervals for designated amount of time. GFP channels were acquired once at the beginning of the time-lapse series and once at the end, to minimize any spurious activation of LOV2-domain built into the iRIS-B system. The optogenetic activation was performed by manually switching on the activation light at a time point between the 60th and the 61st frames. Imaging was continued for 180 frames (30 min) following the optogenetic activation. The morphometric parameters of a cell were calculated following the tracking of cell edge using the Morphodynamics software package^59,60^. Briefly, intensity thresholding was used to construct segmentation masks of the cell images at every time point in time-lapse movies, using the mCherry fluorescence channel. The cell edges were determined automatically from these binary masks at every time point by tracking the position of edges over time by using a previously described software^59,60^ (Supplementary Fig. 8). The cell area within a given region of interest at a given time point in a time series was determined to be the number of positive intensity pixels from the edge of the image frame to that terminating at the tracked edge location per frame. The absolute rate of cell area change was calculated by taking the absolute value of the time differential of the cell area data (this accounted for both expansion and the shrinkage of cell area; thus, characterizing the total ability of a cell to mobilize the cell edge). The data were then averaged within 10 min bins (60 frame differentials) and ratios of experimental (iB(actin) with iRIS-B) to control (iRIS-B only) conditions were calculated within those 10 min bins of average values.

### Reproducibility

The experiments were not randomized. The investigators were not blinded to allocation during the experiments and outcome assessment. No sample-size estimation was performed to ensure adequate power to detect a prespecified effect size.

### Reporting summary

Further information on research design is available in the Nature Research Reporting Summary linked to this article.

## Supplementary information

Supplementary Information

Description of Additional Supplementary Files

Supplementary Movie 1

Supplementary Movie 2

Supplementary Movie 3

Supplementary Movie 4

Supplementary Movie 5

Supplementary Movie 6

Supplementary Movie 7

Supplementary Movie 8

Supplementary Movie 9

Supplementary Movie 10

Reporting Summary

## Data Availability

The main data supporting the findings of this study are available within the article and its Supplementary Materials. The source data underlying Fig. 3d–f, 5b, g, 6c, d, g, 8a, c, and 9b, e, and Supplementary Figs. 1a–f, 3b, c, 4b–g, 11a, b, and 12 are provided as a Source Data file. The additional data are available from the corresponding author on reasonable request.

## Source data

Source Data
