# Supplementary material for: Optogenetic regulation of endogenous proteins
Source: Nat Commun. 2020 Jan 30;11:605. doi: 10.1038/s41467-020-14460-4 (PMC6992714; doi:10.1038/s41467-020-14460-4)
Supplement: Supplementary file 1 — Supplementary Information [file 41467_2020_14460_MOESM1_ESM.pdf]

Optogenetic regulation of endogenous proteins

Taras A. Redchuk, Maksim M. Karasev, Polina V. Verkhusha, Sara K. Donnelly, Maren Hülsemann, Jori Virtanen, Henna M. Moore, Maria K. Vartiainen, Louis Hodgson and Vladislav V. Verkhusha

Supplementary Information

Supplementary Table 1. **Plasmids designed in this study.**

Plasmid	Figure	Insert	Backbone	Addgene number
pQP-2460	1a, b, S1,2	*NcoI*-mVenus-CAAX-IRES2-BphP1-iB(GFP)-IRES2-NES-mCherry-Q-PAS1-*NotI*	pIRES2-EGFP	138973
pQP-2376	S2b	*NcoI*-mVenus-CAAX-IRES2-BphP1-iB(GFP)-IRES2-NSImb-NES-mCh-Q-PAS1-*NotI*	pIRES2-EGFP	138974
pQP-2345	1c-f, 2 S3, 4	*HincII*-EGFP- puromycin N-acetyltransferase-*XbaI*	pPB-Exp (PiggyBac, VectorBuilder)	Not available from Addgene
pQP-2363	1c, d, S1	*NcoI*-BphP1- iB(GFP) -IRES2-NES-mCherry-Q-PAS1-NLS-*NotI*	pIRES2-EGFP	138975
pQP-2473	1e, f, S1	*NcoI*-BphP1-NES-T2A- iB(GFP)-NES-mCherry-Q-PAS1-NLS-*NotI*	pIRES2-EGFP	138976
pQP-NiRISB	2, S4	*NcoI*-BphP1-CAAX-IRES2-NES-iB(GFP)-mCherry-Q-PAS1-AsLOV2cNLS-*NotI*	pIRES2-EGFP	138977
pQP-CiRISB	2, S4	*NcoI*-BphP1-CAAX-IRES2-iB(GFP)-NES-mCherry-Q-PAS1-AsLOV2cNLS-*NotI*	pIRES2-EGFP	138978
pQP-iRISB	2-5, S4-8	*NcoI*-BphP1-CAAX-IRES2-NES-SGlinker-iB(GFP)-mCherry-Q-PAS1-AsLOV2cNLS-*NotI*	pIRES2-EGFP	138979
pQP-iRISBa	3g, 4b	*NcoI*-BphP1-CAAX-IRES2-NES-SGlinker-iB(actin)-mCherry-Q-PAS1-AsLOV2cNLS-*NotI*	pIRES2-EGFP	Not available from Addgene
pQP-AR10	6-9, S9-12	*BglII*-BphP1-NES-T2A- iB(RAS)-NES-mCherry-Q-PAS1-NLS-*NotI*	pIRES2-EGFP	138980

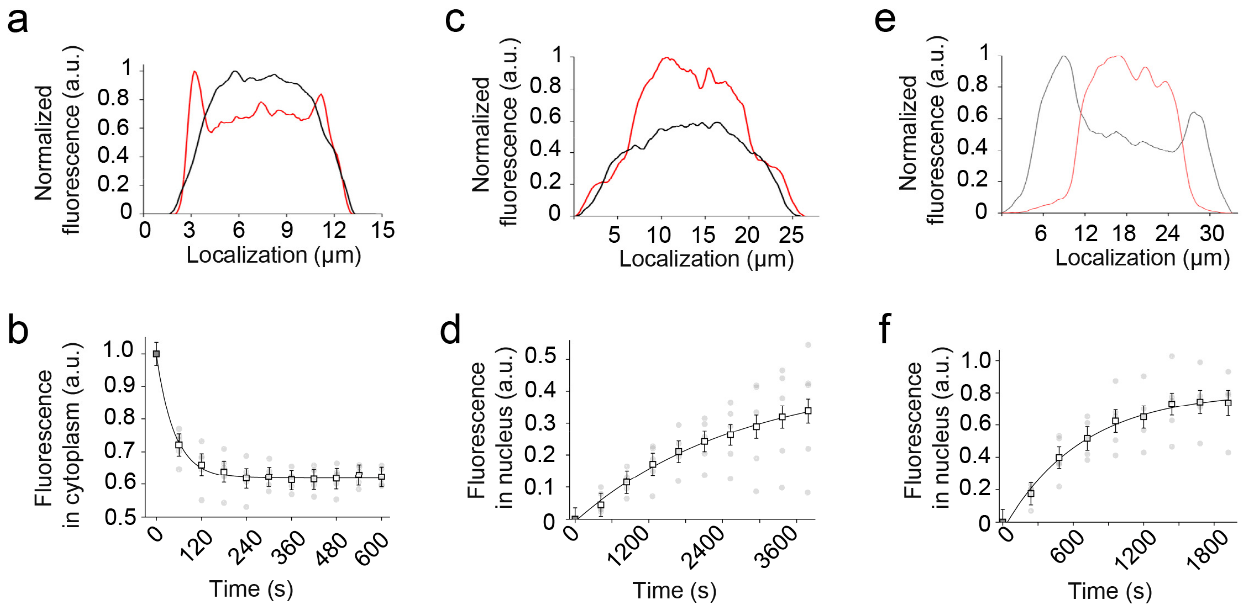

Supplementary Figure 1. Engineering intrabodies to enable their optogenetic control. (**a**) Intensity profiles of mCherry fluorescence of the cell shown in Figure 1b before (black line) and after (red line) 740 nm illumination. (**b**) Kinetics of QPAS1-mCherry depletion in cytoplasm, as detected by mCherry fluorescence. Error bars represent SEM calculated for ROI, dots show individual signal values from 5 quantified cells. (**c**) Intensity profiles of EGFP fluorescence of the cell shown in Figure 1d before (black line) and after (red line) 740 nm illumination. (**d**) Kinetics of EGFP-PAC accumulation in nuclei, due to relocalization of BphP1-iB(GFP), as detected by EGFP fluorescence. Error bars represent SEM calculated for ROI, n = 5 cells. (**e**) Intensity profiles of EGFP fluorescence of the cell in Figure 1f before (red line) and after (black line) 30 min of 740 nm illumination. (**f**) Kinetics of light-triggered EGFP-PAC accumulation in cytoplasm, as detected by EGFP fluorescence. Error bars represent SEM calculated for ROI, n = 5. (**a-f**) Source data are provided as a Source Data file.

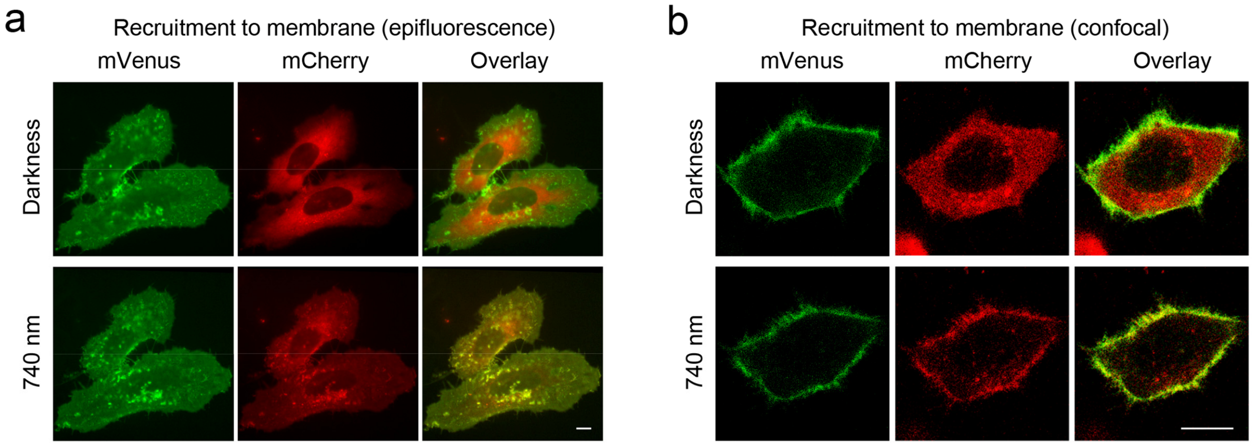

Supplementary Figure 2. (**a**) Representative images showing the NIR-light-controlled recruitment of a protein-of-interest to the plasma membrane, mediated by iB(GFP) interaction with mVenus. In this experiment, HeLa cells transiently expressed BphP1-iB(GFP) fusion, mVenus-CAAX and mCherry-QPAS1. Under NIR light (740 nm), because of the light-controlled interaction of BphP1 and QPAS1, the mCherry-QPAS1 is recruited to the plasma membrane. Epifluorescence microscopy; scale bar, 10 μm. (**b**) NIR light-controlled recruitment of a protein-of-interest to the plasma membrane mediated by iB(GFP) interaction with mVenus, as imaged by confocal microscopy; scale bar, 10 μm.

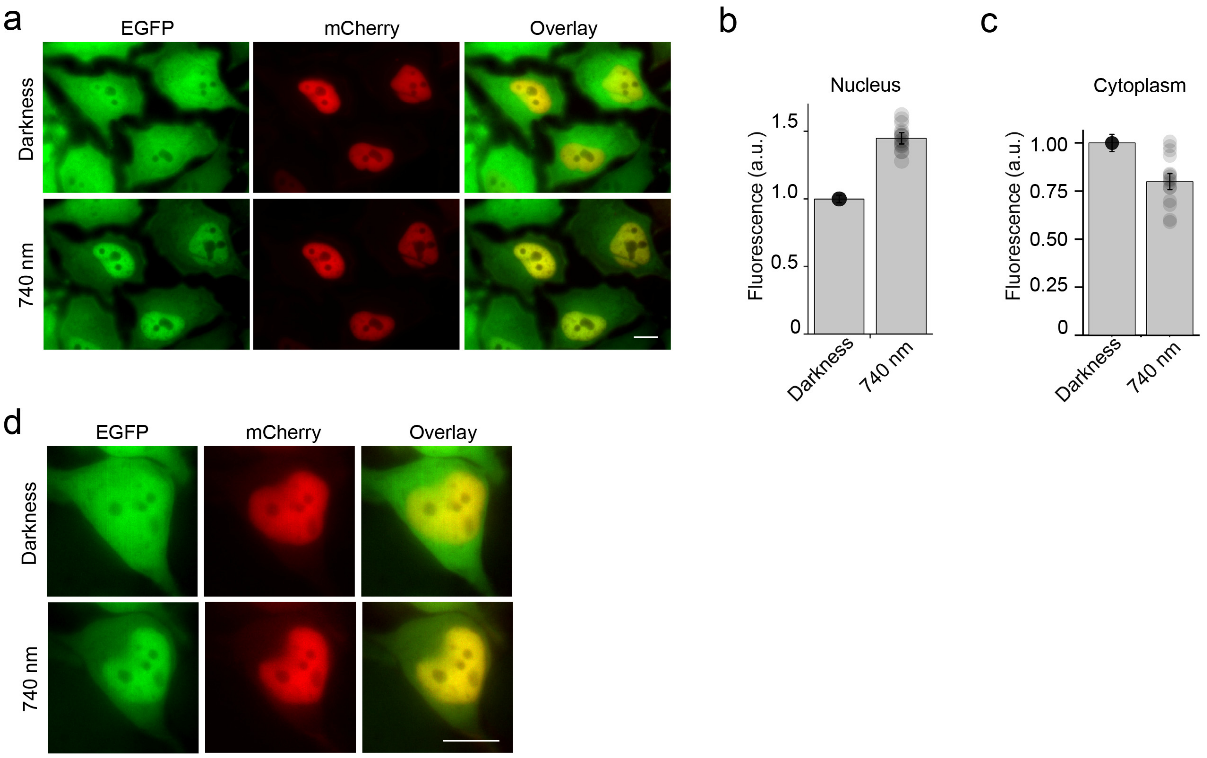

Supplementary Figure 3. Representative images showing genomically expressed EGFP-PAC accumulation in the nucleus, caused by the light-controlled BphP1-iB(GFP) and NES-mCherry-QPAS1-NLS interaction. In darkness, the QPAS1 fusion is shuttling between nucleus and cytoplasm, driven by a strong NLS and a weak NES. Upon 740 nm illumination, it interacts with BphP1 and recruits EGFP-PAC to the nucleus. (**a**) Wide field of view of the sample shown in Figure 1d. (**b, c**) Fluorescence signal changes in nucleus and cytoplasm of the cells shown in (a) averaged over the shown expressing cells (5 regions of interest per cell). Error bars represent SEM, n=3. Source data are provided as a Source Data file. (**d**) Representative cell illustrating performance of the system for cytoplasm-to-nucleus relocalization. Epifluorescence microscopy; scale bar, 10 μm.

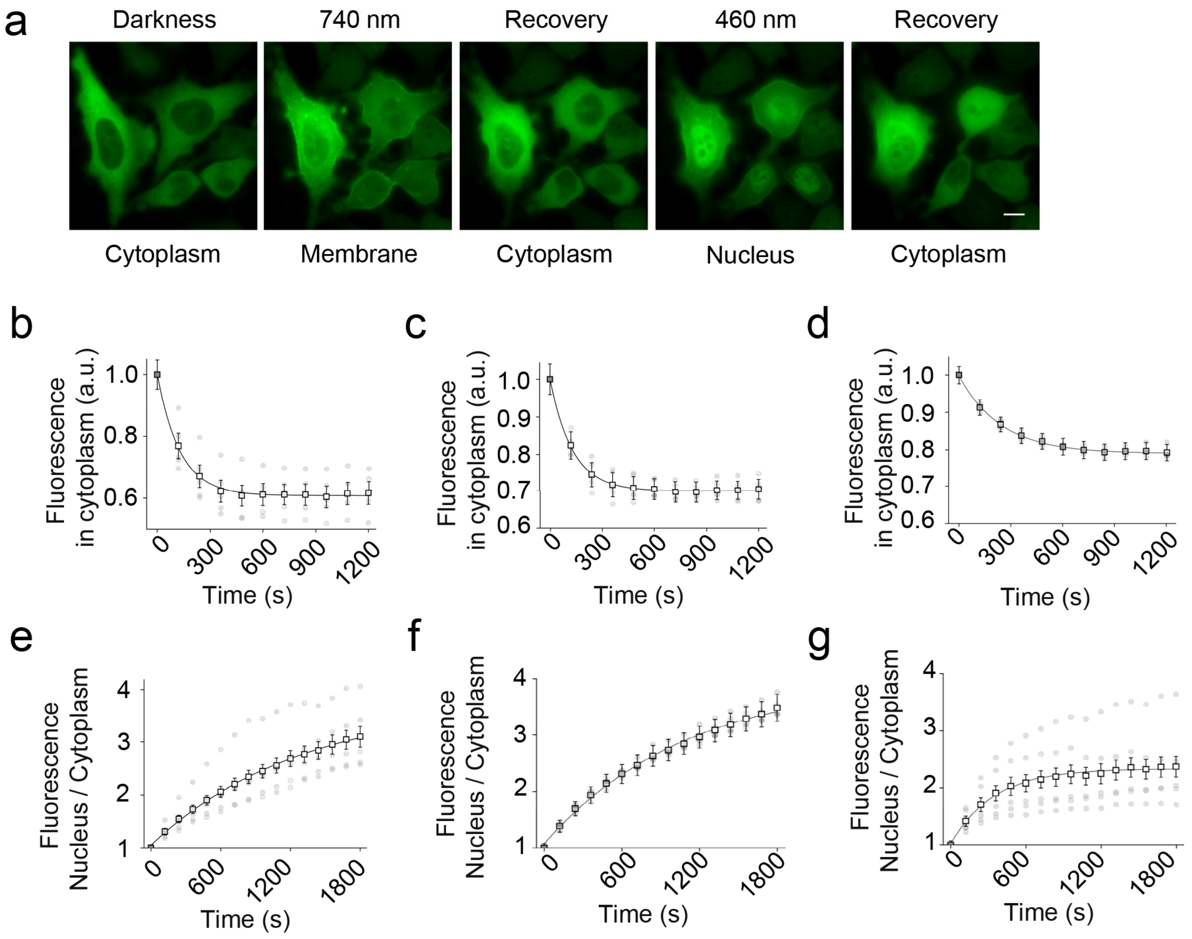

Supplementary Figure 4. Two-color light-controlled relocalization of a genomically expressed target. (**a**) Sequential targeting of EGFP-PAC from cytoplasm to plasma membrane and to nucleus in a cell after irradiation with light of the indicated wavelength and recovery due to thermal relaxation in darkness. The cells were imaged under conditions similar to those in Figure 2b. All five cells in the presented field of view demonstrated the same phenotype dependent on the illumination conditions, showing tolerable cell-to-cell variability and reliable performance of the iRIS-B system. Epifluorescence microscopy; scale bar, 10 µm. (**b, c, d**) Kinetics of EGFP-PAC depletion in cytoplasm under NIR illumination in cells expressing iRIS-B (b), niRIS-B (c) and ciRIS-B (d), error bars represent SEM calculated for ROI, n = 5 cells. Source data are provided as a Source Data file. (**e, f, g**) Kinetics of EGFP-PAC transfer from cytoplasm to the nucleus, in cells expressing iRIS-B (e), niRIS-B (f) and ciRIS-B (g), calculated as the ratio of nuclear to cytoplasmic EGFP intensities, error bars represent SEM calculated for ROI, n = 5 cells. Source data are provided as a Source Data file.

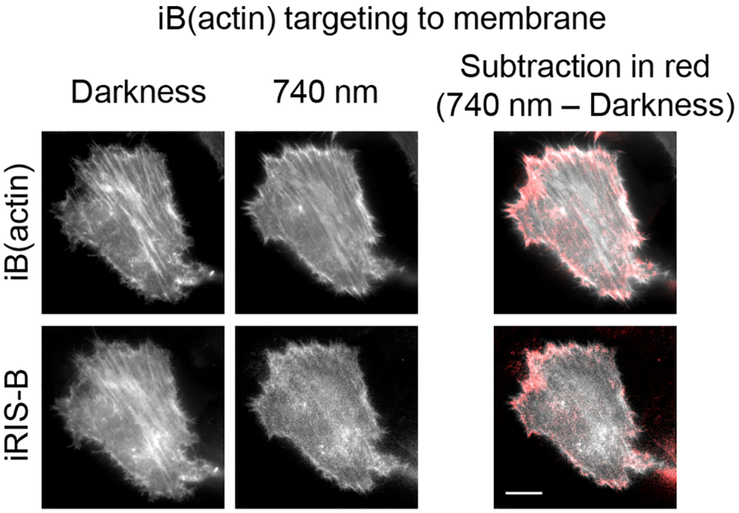

Supplementary Figure 5. Anti-actin intrabody relocalization in iRIS-B-expressing HeLa cells under NIR light. In HeLa cells, co-transfected with iRIS-B and anti-actin intrabody (iB) in darkness intrabody highlights cortical actin, stress fibers and is excluded from the nucleus. Under near-infrared light, iB(actin) disengages from stress fibers and cytoplasmic structures and binds plasma membrane due to interaction with BphP1-CAAX. Subtraction of signal in darkness from signal under illumination is shown on the right in red color, highlighting the regions where the fluorescence signal increased. 16-bit images are analysed, results below zero are set to zero. Epifluorescence microscopy; scale bar, 10 μm.

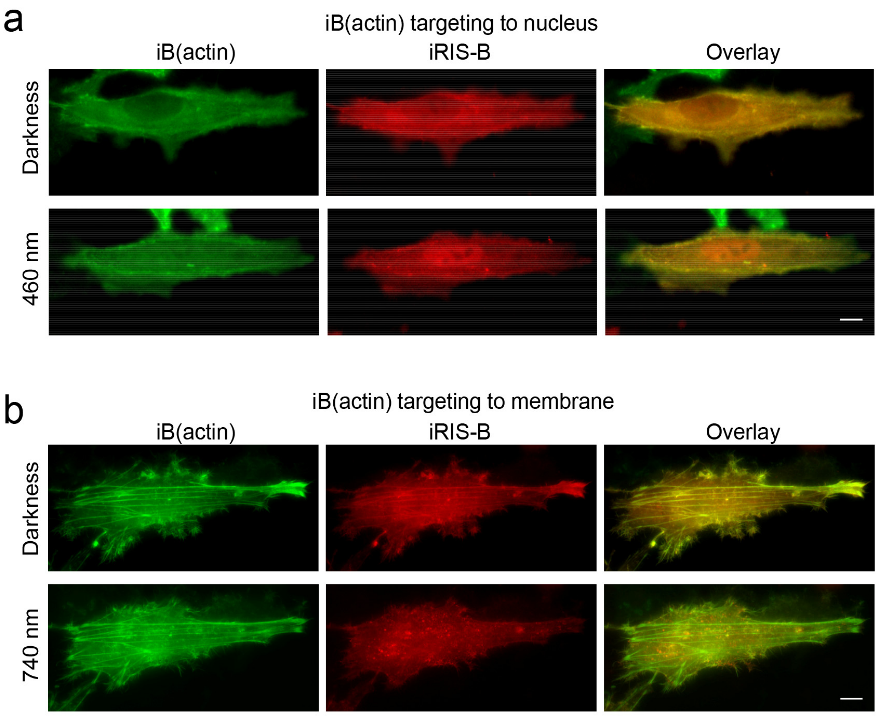

Supplementary Figure 6. Spectral multiplexing of NIR and blue optogenetic systems in iRIS-B enables the tri-directional targeting of a protein-of-interest. (**a**) Representative cell with accumulation of iB(actin) in the nucleus, driven by iRIS-B under 460 nm illumination, is shown. Before illumination, intrabody predominantly highlights stress fibers and is excluded from the nucleus. Under blue light, iB(actin) signal is increased in the nucleus, becoming equal to signal in cytoplasm. Epifluorescence microscopy; scale bar, 10 μm. (**b**) In cells similar to those shown in (a), under 740 nm light, iB(actin) is released from stress fibers and cytoplasmic structures and binds the plasma membrane due to the interaction with BphP1-CAAX. Epifluorescence microscopy; scale bar, 10 μm.

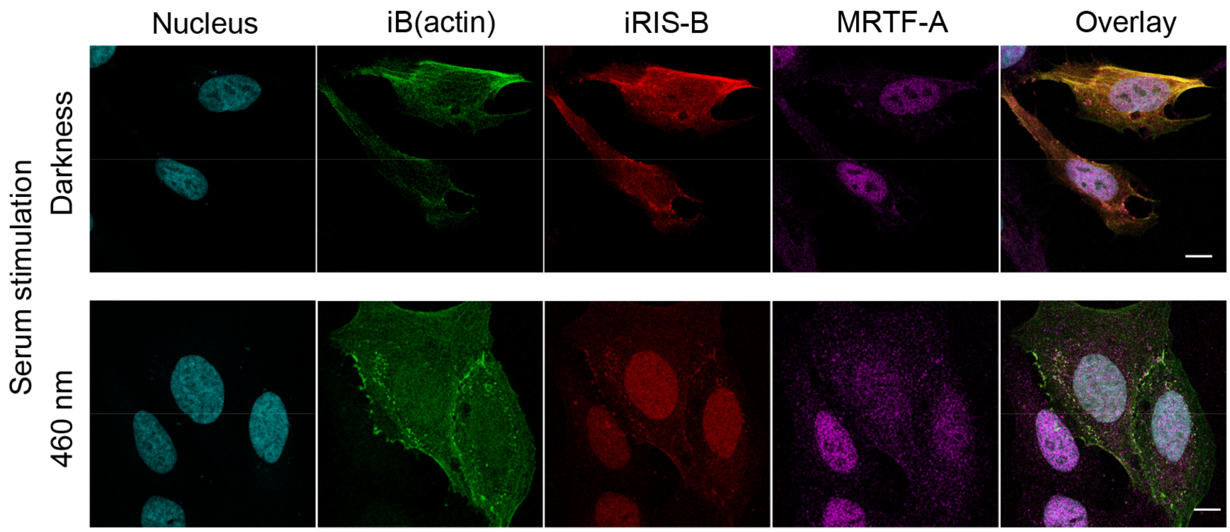

Supplementary Figure 7. Light-dependent nuclear export of MRTF-A in cells co-expressing iRIS-B and iB(actin) under serum stimulation in representative cells. After cell fixation, MRTF-A was visualized using immunostaining. Confocal microscopy; scale bar, 10 μm.

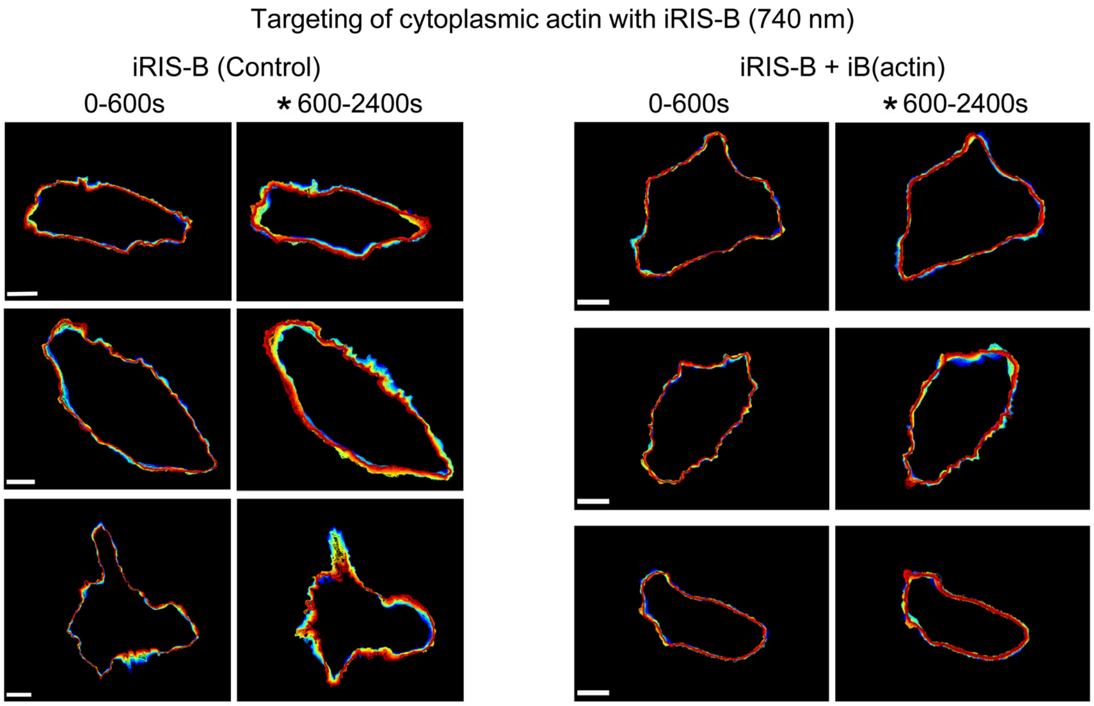

Supplementary Figure 8. Representative edge trace evolutions for iRIS-B control versus iRIS-B + iB(actin)m, with and without 740 nm illumination. Cell edges were tracked using previously described computational routines [1, 2], based on intensity thresholded binary masks produced against mCherry fluorescence. Epifluorescence microscopy; scale bar, 20 µm. Pseudocolor represents elapsed time, from blue to red.

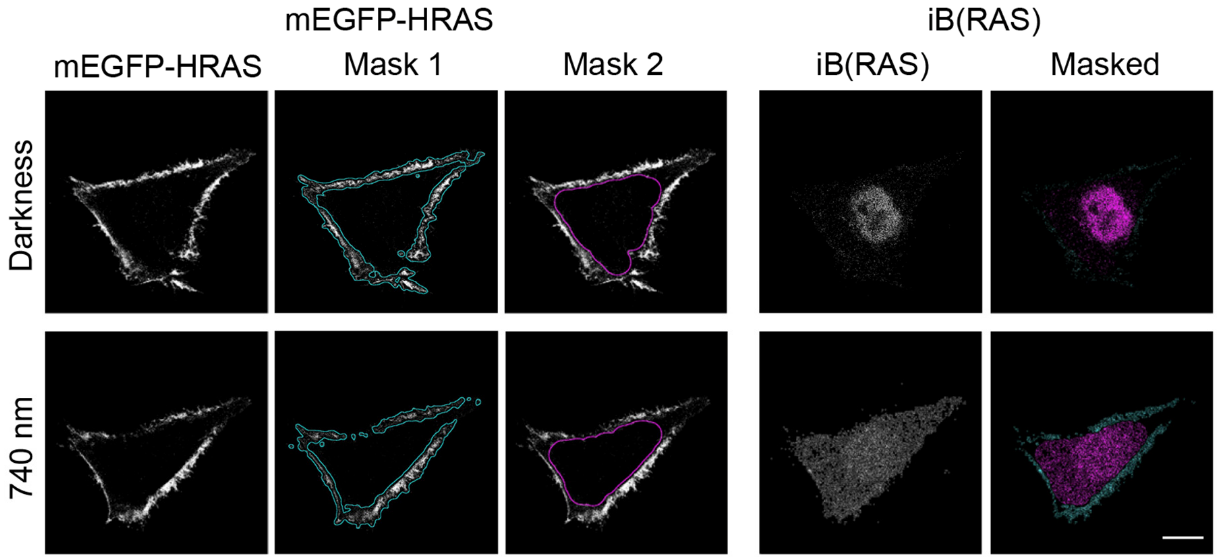

Supplementary Figure 9. Schematics of image analysis used for quantification of fluorescence associated with the plasma membrane. *Left:* mEGFP-HRAS panels are the original images captured in green channel used for setting up the masks highlighting (1) the plasma membrane structures and (2) the inner cell area. Mask #1 (cyan) was obtained by Gaussian blurring with subsequent histogram-based thresholding. Mask #2 outlines the inner cell area (magenta). It was obtained using the analogous steps as Mask #1, with subsequent morphological operations to fill the inner cell area and Mask #1 subtraction. *Right:* iB(RAS) panels show the signal captured in red channel, which was quantified using the binary masks. Pseudocolors in the "Masked" images correspond to those in the left panels. Confocal microscopy; scale bar, 10 µm.

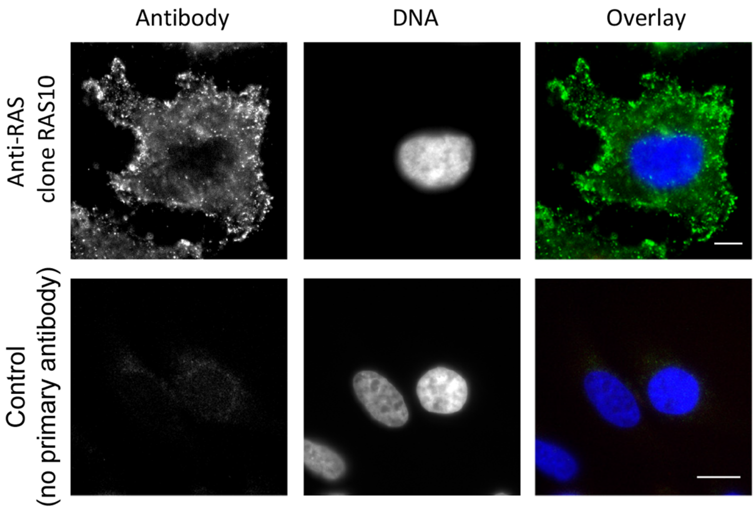

Supplementary Figure 10. RAS staining with anti-pan-RAS monoclonal antibodies in HeLa cells. Non-transfected HeLa cells fixed in PFA and stained with anti-pan-RAS monoclonal antibodies with secondary AlexaFluor 488 conjugated antibody (green). Endogenous RAS can be seen mainly near the cell perimeter. Lower panel represents control cells stained with secondary antibodies only. Images were acquired with equal exposure time. DNA staining is shown in blue. Epifluorescence microscopy; scale bar, 10 μm.

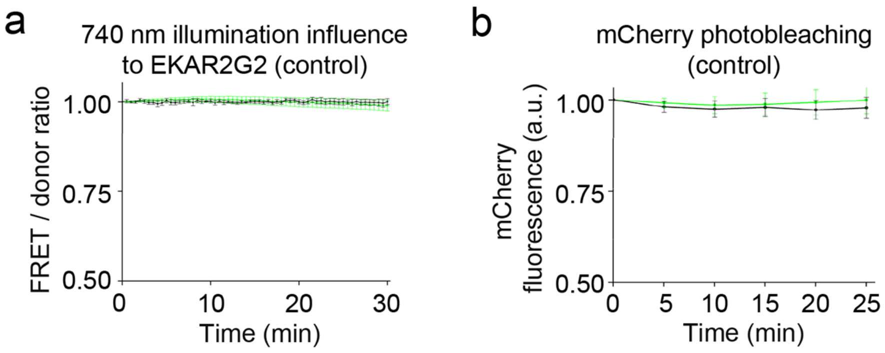

Supplementary Figure 11. Quantification of the time-lapse images from control experiments to assess the unwanted effects of (**a**) 740 nm illumination on EKAR2G2 signal and (**b**) 740 nm light and excitation light during imaging on mCherry fluorescence. Black lines: control cells without light-activation. Green lines: cells illuminated with 740 nm light. In both cases, the unwanted influence was negligible. Error bars represent SEM, n=3 independent experiments. (**a, b**) Source data are provided as a Source Data file.

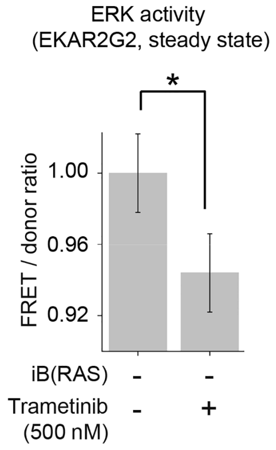

Supplementary Figure 12. Dynamic range of FRET based biosensor for Erk (EKAR2G2) under steady state conditions. Cells were treated for 2 h in 500 nM Trametinib in DMSO, and fixed using 3.7% formaldehyde in PBS for 20 min, followed by imaging. The control condition contained appropriate concentration of DMSO without the inhibitor. n=3 independent experiments with 15 individual cells per experiment. *<0.05 (Student-t test). Error bars represent SEM. Source data are provided as a Source Data file.

Supplementary Note 1. iRIS-B engineering.

Since the position of NES sequence in polypeptide chain may be important for NES signal spatial accessibility and functionality, we assessed the performance of three different variants of NES position in iRIS-B fusion. As expected, tested constructs showed shifted nucleus-to-cytoplasm equilibrium, comparing to original iRIS (**Fig. 2**). A construct with N-terminal NES without a linker in-between NES signal and iB(GFP), named niRIS-B, tend to localize in nucleus (**Fig. 2b middle, Supplementary Fig. 4c, f**), showing no notable recovery after blue illumination. On the contrary, the construct with internal NES sequence iB(GFP)-NES-mCherry-QPAS1-AsLOV2cNLS, named ciRIS-B, showed substantial depletion in the nucleus in darkness and under NIR light, but rather high concentration in cytoplasm under 460 nm light (**Fig. 2b lower, Supplementary Fig. 4c, f)**.

Best performance with sufficient and reversible relocalization under both blue and NIR light was observed for construct designated iRIS-B, bearing N-terminal NES fused to iB(GFP) via flexible 12 a.a. glycine-serine linker. HeLa cells stably expressing EGFP-PAC and transiently expressing iRIS-B showed green fluorescence evenly distributed in cytoplasm (**Fig. 2b upper, Supplementary Fig. 4a**) in darkness. Upon NIR illumination iRIS-B-bound EGFP-PAC moved to plasma membrane (**Supplementary Fig. 4a, b**), while upon blue light (460 nm) the highest concentration of target protein was observed in the nucleus (**Supplementary Fig. 4a, e)**. The NIR light-driven transition of iRIS-bound GFP-fusion was slower than QPAS1-mCherry recruitment to plasma membrane (half-time values are 90 s and 33 s, respectively). On the contrary, relocalization of iRIS-B and EGFP-PAC complex was faster than accumulation of EGFP-PAC in the nucleus, driven by QPAS1-mCherry shuttling (half-time 1444 s *vs*. 1890 s).

These results demonstrate the possibility of multicolor control of endogenous targets localization using optogenetic tools derived from bacterial phytochromes.

Supplementary Note 2. Optically controlled intrabody against RAS.

To enable optical regulation of endogenous RAS, iB(RAS) was fused to mCherry-QPAS1 and tagged with both NLS and NES tags for nuclear-cytoplasmic shuttling shifted to the nucleus (**Fig. 5a**). iB(RAS)-mCherry-QPAS1 construct was placed after NES-tagged BphP1 via T2A, resulting in plasmid named pQP-AR10. In cells transfected with pQP-AR10, mCherry fluorescence was observed in the nucleus (**Fig. 5a, b**). Under 740 illumination, iB(RAS)-mCherry-QPAS1 moved to cytoplasm due to binding to BphP1-NES. The process was relatively quick, the half-time was

516 s (**Fig. 6c**). Further, in darkness iB(RAS)-carrying construct relocalized back to the nucleus, showing the reversibility of the light-controlled regulation. The recovery of nuclear fluorescence was slightly slower than nuclear depletion, half-time was 690 s (**Fig. 6d**). Thus, we designed the construct for effective iB sequestration in the nucleus in darkness and its fast light-triggered release to the cytoplasm, reaching the plasma membrane-associated endogenous RAS under NIR light. For further characterization of optically controlled iB(RAS) performance, immunostaining or RAS was performed in pQP-AR10 expressing cells.

Supplementary Note 3. RAS-Akt signalling in cells expressing optically controlled iB(RAS). In addition to MAPK pathway, RAS also signals through phosphoinositide-3-kinase (PI3K) to activate Akt (protein kinase B) by producing phosphatidylinositol (3,4,5)-trisphosphate (PIP_3), initiated by receptor tyrosine kinases (RTK)[3, 4]. The RAS-Akt pathway is differentially controlled from that of RAS-ERK (**Fig. 9f**), with negative feedback mechanism based on transcriptional regulation of RTK, likely resulting in differential adaptation characteristics to perturbations. To observe the effects of iB(RAS) release on RAS-Akt pathway, we used another FRET biosensor based on cyan-yellow wavelengths, AktAR2, to detect the ability of Akt to phosphorylate its downstream substrate[5-7]. We imaged HeLa cells in serum under steady-state conditions and imaged the AktAR2 while irradiating cells with 740 nm light (**Fig. 9d, Supplementary Movies 8-10**). We saw a loss of FRET/donor ratio during irradiation, indicating that the release of iB(RAS) impacted RAS-Akt pathway as expected. We next illuminated the cells for 2400 s at 740 nm prior to the start of AktAR2 imaging and measured the FRET/donor ratio during the dark relaxation. In contrast to RAS-ERK pathway, the ability of Akt to phosphorylate the substrate did not recover during the period of dark relaxation (**Supplementary Fig. 9d, e**). This suggested that RAS-Akt pathway may require a significantly longer time scale to re-attain the homeostatic equilibrium following perturbation.

Supplementary Note 4. Optical regulation of RAS-ERK and RAS-Act sub-networks.
The endogenous signaling pathways targeted for optical modulation herein were two pathways regulated by RAS GTPase: The MAPK pathway, traditionally known to be initiated by growth factor receptors activating Grb2-Sos complex which acts on p21 small GTPase RAS, inducing the classic kinase cascade that culminates in activation of ERK (**Fig. 9**) to drive transcriptional

regulation[8, 9]; and the PI3K pathway, initiated by RTK signaling through RAS, inducing activation of Akt which drives many number of downstream transcriptional programming[10, 11] (**Fig. 9**). The FRET biosensor readouts from these two pathways suggested similar ability of our QPAS1-iB(RAS) to inhibit RAS when optogenetically released. We showed however, that the abilities of these two signaling pathways to recover during the dark relaxation were clearly different. The ERK pathway appeared to be more rapidly re-tunable following perturbation, owing likely to the feedback control of this pathway at the activity modulation of intermediate kinases within the normal signaling cascade instead of the transcriptional regulation of signaling components which would require longer time scales for adaptation[8]. In the case of RAS-Akt pathway, the optogenetic perturbation was not well recovered within our imaging experiments, suggesting longer time scales may be necessary. This may point to the control mechanism of RAS-Akt pathway that depends more on the transcriptional-level of regulation[8, 10, 11]. It is also possible however, that Akt could be directly impacted by other pathways in addition to RAS, including PDK1, mTORC2 and protein phosphatases[12-18]. Together, these additional pathways may impact strongly the ability of Akt to re-tune and attain homeostatic equilibrium following optogenetic perturbation.

Supplementary References

1. Machacek, M. et al. Coordination of Rho GTPase activities during cell protrusion. *Nature* **461**, 99-103 (2009).
2. Machacek, M. & Danuser, G. Morphodynamic Profiling of Protrusion Phenotypes. *Biophys. J.* **90**, 1439-1452 (2006).
3. Castellano, E. & Downward, J. Role of RAS in the regulation of PI 3-kinase. *Current topics in microbiology and immunology* **346**, 143-169 (2010).
4. Castellano, E. & Downward, J. RAS Interaction with PI3K: More Than Just Another Effector Pathway. *Genes & cancer* **2**, 261-274 (2011).
5. Gao, X. & Zhang, J. Spatiotemporal analysis of differential Akt regulation in plasma membrane microdomains. *Mol Biol Cell* **19**, 4366-4373 (2008).
6. Gao, X. & Zhang, J. Akt signaling dynamics in plasma membrane microdomains visualized by FRET-based reporters. *Commun Integr Biol* **2**, 32-34 (2009).
7. Zhou, X. et al. Dynamic Visualization of mTORC1 Activity in Living Cells. *Cell Rep* **10**, 1767-1777 (2015).
8. Johnson, H.E. et al. The Spatiotemporal Limits of Developmental Erk Signaling. *Dev Cell* **40**, 185-192 (2017).
9. Fanger, G.R., Gerwins, P., Widmann, C., Jarpe, M.B. & Johnson, G.L. MEKKs, GCKs, MLKs, PAKs, TAKs, and tpls: upstream regulators of the c-Jun amino-terminal kinases? *Curr Opin Genet Dev* **7**, 67-74 (1997).
10. Manning, B.D. & Toker, A. AKT/PKB Signaling: Navigating the Network. *Cell* **169**, 381-405 (2017).
11. Aksamitiene, E., Kiyatkin, A. & Kholodenko, B.N. Cross-talk between mitogenic Ras/MAPK and survival PI3K/Akt pathways: a fine balance. *Biochem Soc Trans* **40**, 139-146 (2012).
12. Xie, X. et al. IkappaB kinase epsilon and TANK-binding kinase 1 activate AKT by direct phosphorylation. *Proc Natl Acad Sci U S A* **108**, 6474-6479 (2011).
13. Sarbassov, D.D., Guertin, D.A., Ali, S.M. & Sabatini, D.M. Phosphorylation and regulation of Akt/PKB by the rictor-mTOR complex. *Science* **307**, 1098-1101 (2005).
14. Di Maira, G., Brustolon, F., Pinna, L.A. & Ruzzene, M. Dephosphorylation and inactivation of Akt/PKB is counteracted by protein kinase CK2 in HEK 293T cells. *Cell Mol Life Sci* **66**, 3363-3373 (2009).
15. Chan, C.H. et al. Posttranslational regulation of Akt in human cancer. *Cell Biosci* **4**, 59 (2014).
16. Liu, P., Wang, Z. & Wei, W. Phosphorylation of Akt at the C-terminal tail triggers Akt activation. *Cell Cycle* **13**, 2162-2164 (2014).
17. Dan, H.C., Antonia, R.J. & Baldwin, A.S. PI3K/Akt promotes feedforward mTORC2 activation through IKKalpha. *Oncotarget* **7**, 21064-21075 (2016).
18. Sathe, A. et al. Parallel PI3K, AKT and mTOR inhibition is required to control feedback loops that limit tumor therapy. *PLoS One* **13**, e0190854 (2018).
