# Supplementary material for: Optogenetic regulation of endogenous proteins
Source: Nat Commun. 2020 Jan 30;11:605. doi: 10.1038/s41467-020-14460-4 (PMC6992714; doi:10.1038/s41467-020-14460-4)
Supplement: Supplementary file 2 — Description of Additional Supplementary Files [file 41467_2020_14460_MOESM2_ESM.pdf]

Description of Additional Supplementary Files

File Name: Supplementary Movie 1
Description: NIR light control of cell motility in cells expressing iRIS-B and anti-actin intrabody, iB(actin). Representative HeLa cell from the sample used for quantification shown in Figure 4f, transfected with iRIS-B and iB(actin). In darkness before illumination, mCherry-iRIS-B localized similar to F-actin distribution patterns visualized through the iB(actin) GFP channel. Upon 740 nm light, at 10 min into live cell imaging, the cellular distribution of mCherry-iRIS-B shifted from the Factin-like to a punctated distribution that is reminiscent of plasma membrane localization. Reduction in the rate of cell area change was shown in cells under NIR light. Scale bar, 10 µm. Frame rate: 7 fps, time-lapse imaging rate: 10 s intervals.

File Name: Supplementary Movie 2
Description: NIR light control of cell motility in cells expressing iRIS-B, no-intrabody control. Representative control HeLa cell from the sample used for quantification shown in Figure 4f, transfected with iRIS-B only, without anti-actin intrabody. Under 740 nm illumination at 10 min into live cell imaging, the cellular distribution of mCherry-iRIS-B changed from the even cytoplasmic to plasma membrane-localized. No changes in cell motility were observed. Scale bar, 10 µm. Frame rate: 7 fps, time-lapse imaging rate: 10 s intervals.

File Name: Supplementary Movie 3
Description: Representative edge tracking traces for control HeLa cells expressing only the iRIS-B, undergoing 740nm illumination (indicated by a red asterisk; turned on at 10 min until the end of imaging). Scale bar, 20 µm. Frame rate: 14 fps, time-lapse imaging rate: 10 s intervals.

File Name: Supplementary Movie 4
Description: Representative edge tracking traces for HeLa cells expressing both the iRISB and iB (actin), undergoing 740nm illumination (indicated by a red asterisk; turned on at 10 min until the end of imaging). Scale bar, 20 µm. Frame rate: 14 fps, time-lapse imaging rate: 10 s intervals.

File Name: Supplementary Movie 5
Description: RAS-ERK downregulation by optically-triggered anti-RAS intrabody, iB(RAS), uncaging. Timelapse video of EKAR2G1 FRET/donor ratio, imaged in HeLa cells in serum. 740 nm illumination was applied at 300 s time point (white asterisk) and remained on thereafter until the end of an experiment. Scale bar, 20 µm. Pseudocolor scale: black = 1.0; white = 2.05. Frame rate: 7 fps, time-lapse imaging rate: 10 s intervals.

File Name: Supplementary Movie 6
Description: Recovery of RAS-ERK signalling in cells expressing optically-controlled iB(RAS) kept in darkness after NIR illumination. For dark recovery experiments 740 nm illumination was applied for 2400 s prior to the start of EKAR2G1 imaging, and turned off at 0 s time point and remained off for the duration of an experiment as shown. Scale bar, 20 µm. Pseudocolor scale: black = 1.0; white = 2.05. Frame rate: 7 fps, time-lapse imaging rate: 10 s intervals.

File Name: Supplementary Movie 7
Description: RAS-ERK signalling in cells expressing optically-controlled intrabody kept in darkness (control). No measurable changes of RAS signalling activity, as followed by EKAR2G1 imaging, were observed. Scale bar, 20 µm. Pseudocolor scale: black = 1.0; white = 2.05. Frame rate: 7 fps, time-lapse imaging rate: 10 s intervals.

File Name: Supplementary Movie 8
Description: RAS-Akt downregulation by optically-controlled iB(RAS). Timelapse video of AktAR2 FRET/donor ratio, imaged in HeLa cells in serum. 740 nm illumination was applied at 300 s time point (white asterisk) and remained on thereafter until the end of an experiment. Scale bar, 20 µm. Pseudocolor scale: black = 1.0; white = 2.88. Frame rate: 7 fps, time-lapse imaging rate: 10 s intervals.

File Name: Supplementary Movie 9
Description: Recovery of RAS-Akt signalling in cells expressing optically-controlled iB(RAS) kept in darkness after NIR illumination. For dark recovery experiments 740 nm illumination was applied for 2400 s prior to the start of AktAR2 imaging, and turned off at 0 s time point and remained off for the duration of an experiment as shown. Scale bar, 20 µm. Pseudocolor scale: black = 1.0; white = 2.88. Frame rate: 7 fps, time-lapse imaging rate: 10 s intervals.

File Name: Supplementary Movie 10
Description: RAS-Akt signalling in cells expressing optically-controlled intrabody kept in darkness (control). No measurable changes of RAS signalling, as followed by AktAR2 imaging, activity were observed. Scale bar, 20µm. Pseudocolor scale: black = 1.0; white = 2.88. Frame rate: 7 fps, time-lapse imaging rate: 10 s intervals.
