# Supplementary material for: Optogenetic regulation of endogenous proteins
Source: Nat Commun. 2020 Jan 30;11:605. doi: 10.1038/s41467-020-14460-4 (PMC6992714; doi:10.1038/s41467-020-14460-4)
Supplement: Supplementary file 13 — Reporting Summary [file 41467_2020_14460_MOESM13_ESM.pdf]

nature research

Corresponding author(s): Vladislav Verkhusha

Last updated by author(s): 23/12/19

Reporting Summary

Nature Research wishes to improve the reproducibility of the work that we publish. This form provides structure for consistency and transparency in reporting. For further information on Nature Research policies, see Authors & Referees and the Editorial Policy Checklist.

Statistics

For all statistical analyses, confirm that the following items are present in the figure legend, table legend, main text, or Methods section.

n/a	Confirmed	
☐	☒	The exact sample size (*n*) for each experimental group/condition, given as a discrete number and unit of measurement
☐	☒	A statement on whether measurements were taken from distinct samples or whether the same sample was measured repeatedly
☐	☒	The statistical test(s) used AND whether they are one- or two-sided<br>*Only common tests should be described solely by name; describe more complex techniques in the Methods section.*
☒	☐	A description of all covariates tested
☒	☐	A description of any assumptions or corrections, such as tests of normality and adjustment for multiple comparisons
☐	☒	A full description of the statistical parameters including central tendency (e.g. means) or other basic estimates (e.g. regression coefficient) AND variation (e.g. standard deviation) or associated estimates of uncertainty (e.g. confidence intervals)
☐	☒	For null hypothesis testing, the test statistic (e.g. *F*, *t*, *r*) with confidence intervals, effect sizes, degrees of freedom and *P* value noted<br>*Give P values as exact values whenever suitable.*
☒	☐	For Bayesian analysis, information on the choice of priors and Markov chain Monte Carlo settings
☒	☐	For hierarchical and complex designs, identification of the appropriate level for tests and full reporting of outcomes
☒	☐	Estimates of effect sizes (e.g. Cohen's *d*, Pearson's *r*), indicating how they were calculated

Our web collection on statistics for biologists contains articles on many of the points above.

Software and code

Policy information about availability of computer code

Data collection	Microscopy images acquisition was performed using SlideBook v. 6.0.8 (Intelligent Imaging Innovations),
Data analysis	The microscopy data were analyzed using a SlideBook v. 6.0.8 (Intelligent Imaging Innovations), ImageJ v. 1.50b (NIH) and Morphodynamics [Machacek, M. & Danuser, G., 2006] software. NumPy, SciPy and Matplotlib were used as a part of Anaconda python distribution (v. 2019.03). The Flow cytometry data were analyzed using a FlowJo v.7.6.2 software

For manuscripts utilizing custom algorithms or software that are central to the research but not yet described in published literature, software must be made available to editors/reviewers. We strongly encourage code deposition in a community repository (e.g. GitHub). See the Nature Research guidelines for submitting code & software for further information.

Data

Policy information about availability of data

All manuscripts must include a data availability statement. This statement should provide the following information, where applicable:

- Accession codes, unique identifiers, or web links for publicly available datasets
- A list of figures that have associated raw data
- A description of any restrictions on data availability

The main data supporting the findings of this study are available within the article, its Supplementary materials and Source Data file. The additional data are available from the corresponding author on reasonable request. Plasmids constructed during this research, as well as respective maps and sequences, will be deposited in Addgene depository, and accession number will be provided before publication.

Field-specific reporting

Please select the one below that is the best fit for your research. If you are not sure, read the appropriate sections before making your selection.

☒ Life sciences ☐ Behavioural & social sciences ☐ Ecological, evolutionary & environmental sciences

For a reference copy of the document with all sections, see nature.com/documents/nr-reporting-summary-flat.pdf

Life sciences study design

All studies must disclose on these points even when the disclosure is negative.

Sample size	No sample-size calculations were performed. For most of relocalization experiments, unless noted in the figure legend, fluorescent signal was calculated for 5 or more cells, pooled from at least 3 independent experiments. For cell motility studies, 29-35 protrusions were analyzed from 3-4 independent experiments. For fixed cells analysis in nuclear actin functions experiments 14-35 cells were imaged and analyzed.
Data exclusions	No data were excluded from analysis.
Replication	All attempts at replication were successful.
Randomization	The experiments were not randomized.
Blinding	The investigators were not blinded.

Reporting for specific materials, systems and methods

We require information from authors about some types of materials, experimental systems and methods used in many studies. Here, indicate whether each material, system or method listed is relevant to your study. If you are not sure if a list item applies to your research, read the appropriate section before selecting a response.

Materials & experimental systems

n/a	Involved in the study	
☐	☒	Antibodies
☐	☒	Eukaryotic cell lines
☒	☐	Palaeontology
☒	☐	Animals and other organisms
☒	☐	Human research participants
☒	☐	Clinical data

Methods

n/a	Involved in the study	
☒	☐	ChIP-seq
☐	☒	Flow cytometry
☒	☐	MRI-based neuroimaging

Antibodies

Antibodies used	Anti-Pan-Ras antibody, mouse monoclonal IgG2aκ, clone RAS 10 (MABS195, Merck)<br>secondary Alexa Fluor 488-conjugated antibody, anti-mouse goat IgG (A-11001, ThermoFisher)<br>MRTF-A antibody (G-8, Santa Cruz Biotechnology)<br>secondary anti-mouse Alexa Fluor 647 (A-21235, ThermoFisher)
Validation	Both Anti-Pan-Ras and anti-MRTF-A were validated for immunocytochemistry, according the data from manufacturer.

Eukaryotic cell lines

Policy information about cell lines

Cell line source(s)	HeLa cells were purchased from ATCC.
Authentication	No additional authentication was performed for cells purchased in ATCC. ATCC authenticates cell lines using STR analysis, according to the product specification.
Mycoplasma contamination	Cell lines were not tested for mycoplasma.
Commonly misidentified lines (See ICLAC register)	No commonly misidentified cell lines were used.

Flow Cytometry

Plots

Confirm that:

☒ The axis labels state the marker and fluorochrome used (e.g. CD4-FITC).

☒ The axis scales are clearly visible. Include numbers along axes only for bottom left plot of group (a 'group' is an analysis of identical markers).

☐ All plots are contour plots with outliers or pseudocolor plots.

☒ A numerical value for number of cells or percentage (with statistics) is provided.

Methodology

Sample preparation	Cell lines purchased in ATCC were used
Instrument	BD Accuri C6 flow cytometer
Software	CFlow Plus, FlowJo v.7.6.2
Cell population abundance	Not applicable, since sorting was not performed.
Gating strategy	FSC vs SSC gating for distinguishing of cell populations and excluding the cellular debris. Resulted population were analyzed on SSC-A/FL plot to find cells expressing mCherry. Both non-transfected cells and mock transected cells were used for selecting negative population.

☐ Tick this box to confirm that a figure exemplifying the gating strategy is provided in the Supplementary Information.
